# Contrasting holistic-compensatory with probabilistic heuristic strategies in multi-attribute decisions

**DOI:** 10.3758/s13423-025-02795-2

**Published:** 2026-02-27

**Authors:** Gal Atun, Vincent de Gardelle, Marius Usher

**Affiliations:** 1 School of Psychological Sciences, Tel-Aviv University, Haim Levanon 55, Tel Aviv, Israel; 2 Centre d’Economie de la Sorbonne, Université Paris 1, Paris, France; 3 Paris School of Economics and CNRS, Paris, France

**Keywords:** Weighted-average, TTB heuristics, Selective sampling, Choice RT, Computational modeling, Decision-polarization

## Abstract

Recent research on multi-attribute decision-making has challenged the view that in open-view conditions, under time pressure, humans mostly rely on simplified strategies that only examine part of the choice information, as in Take the Best (*TTB*) or the priority heuristics. Here we examine and test a probabilistic extension of *TTB* which preserves the central heuristic idea that each decision is made based on a single attribute but selects this attribute probabilistically (rather than deterministically as in *TTB*) and maintains choice accuracy at levels found in human data. We show that this single probabilistic attribute (*SPA*) model produces choice patterns similar to the compensatory (normative) weighted-average (*WAV*) model, and we computationally compare the *SPA* model with a similar model called *gTTB* (Bergert & Nosofsky, *Journal of Experimental Psychology: Learning, Memory, and Cognition,* 33:107, 2007), showing that *SPA* provides better fit for 3 attributes to choice data (and about equal fit for 4 and 5 attributes). We then show that the heuristic (SPA/gTTB) and compensatory (WAV) models can be distinguished based on decision times, by contrasting high vs. low choice-polarization trials. To arbitrate between the *SPA* and the normative model, we collected data on a speeded multi-attribute decision task with 3, 4 and 5 numerical attributes, in a main and a replication experiment (total *N* = 117 participants). Our data shows significant individual differences in decision strategy. While about 30% of the participants appear to deploy a *TTB* strategy, the majority (70%) show choices that are consistent with either the *SPA* or the WAV models. Contrary to Bergert and Nosofsky (Bergert & Nosofsky, *Journal of Experimental Psychology: Learning, Memory, and Cognition,* 33:107, 2007), we found that the examination of decision-time provided strong evidence against the *SPA* model and supported instead the normative weighted-average account: when presented with choice information in free view most participants were able to carry out fast (mean-RT < 1.5 s) and compensatory decisions that attend to (and weight) all choice attributes.

**Supplementary Information:**

The online version contains supplementary material available at 10.3758/s13423-025-02795-2.

## Introduction

Multi-attribute decision-making, as illustrated in choosing between consumer products, between apartments, or between job candidates, is a complex task that often involves trade-offs between attributes that are incommensurable: one apartment might be cheaper but further away from work compared with another, one candidate might have better recommendation letters but a less pleasant personality than another. The normative theory (e.g., Keeney & Raiffa, 1976) for such decisions involves evaluating each option on the relevant attributes, assigning weights to each attribute and choosing the option with the highest *weighted-average* (*WAV*). This algorithm is characterized as *compensatory*, as, for a given option, a low score on one attribute can be compensated by a high score on another.

A prevailing view, however, rooted in bounded rationality (Gigerenzer, 2004; Payne et al., 1993; Simon, 1955) is that individuals often use simple strategies that minimize the amount of information considered and the mental effort invested in a decision, in particular in speeded conditions with complex decision criteria (see also Ariely & Zakay, 2001; Bettman et al., 1998; Oh et al., 2016; Payne et al., 1988, 1992; Rieskamp & Hoffrage, 1999, 2008). As stated by Payne et al. (1988), in one of the foundational studies for this approach, “People may use heuristics under time pressure, because they have no other choice (Simon, 1981). A more normative decision strategy like utility maximization, may exceed the processing capacity of the decision maker, given any reasonable time limit” (p. 535). To support this statement, Payne et al. have shown that heuristics models achieve a major reduction in elementary information processing steps (*EIP*; which they take as a proxy for mental effort), with only marginal decline in choice accuracy. They then provided empirical support for an adaptive use of heuristics (e.g., heuristics being used more under time pressure) by examining how individuals reveal information in a multi-attribute task where attribute values are initially hidden (this uses the Mouselab tracing method; see, e.g., Willemsen & Johnson, 2011).

Along these lines, a number of studies have demonstrated that when facing complex decisions (with multiple attributes that exert a search or integration cost; Pachur, 2022), especially under time pressure, humans shift to heuristic non-compensatory strategies to save the effort required to carry out compensatory strategies, such as weighted averaging (Ariely & Zakay, 2001; Bettman et al., 1998; Einhorn, 1970; Gigerenzer & Gaissmaier, 2011; Oh et al., 2016; Pachur, 2022; Payne et al., 1988, 1992; Rieskamp & Hoffrage, 1999, 2008). For instance, in quiz questions, where one should search for relevant information in memory (Which of two German cities, Hamburg or Düsseldorf, is more populated?), individuals appear to rely on a single cue (e.g., choose Hamburg because it has a football team in the Bundesliga; Gigerenzer & Goldstein, 1996; Gigerenzer, et al., 1991). In risky choice, where the probabilities and outcomes associated with several risky options are simultaneously presented, triggering conflict between attributes across options, a *priority* heuristic was proposed, in which individuals make a sequence of comparisons between the alternatives, one attribute at a time (Brandstätter et al., 2006), rather than engaging in weighting and summing of values and probabilities.^1^ In multi-attribute decisions, one such heuristic is the lexicographic semi-order strategy, also called the *take-the-best* strategy (hereafter, *TTB*). In *TTB* individuals do not combine all attributes but only consider the most important one (and in case of a tie on this attribute, the second most important, etc.), avoiding thus the need to carry effortful weighted averaging (Fishburn, 1974; Gigerenzer, 2004; Gigerenzer & Goldstein., 1996; Gigerenzer et al., 1991; Payne et al., 1993; Tversky, 1969). Such a strategy is particularly efficient in decision environments where one attribute is highly predictive of the correct answer, and individuals indeed seem able to adjust their strategy depending on the structure of the information in the environment (e.g., Bröder, 2003; Glöckner et al., 2014; Payne et al., 1988; Rieskamp & Otto, 2006).

It is important to note, however, that whereas such non-compensatory heuristics offer “satisfying” solutions that generally work well (Gigerenzer, 2004; Simon, 1955), they also have costs, as they lead to choice patterns that are incompatible with rationality principles, such as violations of preference transitivity and framing effects (Glickman et al., 2018; Shafir, 1993; Tsetsos et al., 2012, 2016; Tversky, 1969; see Regenwetter et al., 2011, and Ranyard et al., 2020, for a reappraisal of the transitivity violation literature).

More recently, a number of studies have also shown that individuals can deploy compensatory strategies even in complex speeded decisions (Ayal & Hochman, 2009; Brusovansky et al., 2018; Bröder, 2000; Glöckner & Betsch, 2008a, 2012; Newell & Shanks, 2003; for a recent review, see Pachur, 2022). For example, in a seminal study of multi-cue probabilistic inference,^2^ Glöckner and Betsch (2008a) have shown, by observing choices, response times and confidence, that participants do not use *TTB* but mostly apply a weighted average strategy (*WAV*),^3^ and can do so very rapidly (in about 1.5 s), provided that all the information is presented (for open view) at once. They then proposed that the data pattern (in choice, RT, and confidence) is best explained by an automatic decision process, in which all the information enters a parallel constraint satisfaction model (*PCS*), which is a recurrent neural network linking the attributes with the option representations, capable of implementing fast and normative decisions (Glöckner & Betsch, 2008b). Supporting the notion of parallel processing, Glöckner and Betsch’s subsequent research has shown that adding a new attribute (with extra information) to the decision problem can shorten response times (rather than slow down responses, as expected if attributes were processed sequentially), if the added information increases the overall information coherence (Glöckner & Betsch, 2012). The idea that observers are capable of deploying rapid compensatory strategies when facing complex decision problems has also received support in situations involving multi-attribute decisions with numerical values (rather than binary cues indicating positive/negative recommendations). For example, in Brusovansky et al. (2018), participants had limited time to select the best option in a pair (of job candidates), where each option was characterized by numerical values on 3, 4 or 5 attributes with given (prescribed) weights. The results showed that the participants can achieve high accuracy (about 85% correct, outperforming the *TTB* heuristic) at a fast speed (mean RT < 2 s) and without any speed–accuracy trade-offs.^4^ Furthermore, for the majority (about 60%) of participants, choices were better explained by *WAV* than by the *TTB* heuristic.

Here, we consider the possibility that *WAV*-like choices (in the study above) may actually stem from a non-compensatory process that merely appears compensatory. Specifically, individuals may still use a single attribute at a time, but select it probabilistically from trial to trial, based on its importance, rather than following a strict lexicographical order as in *TTB*. Critically though, such a probabilistic version of *TTB* might still be distinguished from *WAV* decisions if one examines response times. In a prior study, Bergert and Nosofsky (2007) investigated this issue in a probabilistic learning and inference task, where participants had to learn the predictive value of binary cues through trial-by-trial feedback. In this situation, they found support for a generalized, probabilistic version, of the *TTB* strategy, which they called *gTTB*. In the present work, we use a similar approach but consider a different experimental situation—a speeded multi-attribute decision task, in which the cues take numerical values and have prescribed weights that are transparently presented and do not need to be learned (Brusovansky et al., 2018). A priori, this situation leaves open the possibility for both compensatory and non-compensatory strategies: on the one hand, all the information is immediately visible, while on the other hand, it is also more complex to process (calculating a weighted average is more demanding than counting binary cues) and participants are asked to make their decisions under time pressure.

The present paper is organized as follows. First, we present simulations showing that a strategy, using a single attribute selected in a probabilistic manner, can produce multi-attribute choices that mimic a compensatory strategy. We refer to this as the *single probabilistic attribute* (*SPA*) model (see also Vanunu et al., 2020, for a recent selective sampling model with similar characteristics^5^). We illustrate the limit of this process in terms of choice accuracy, and we introduce a modification to this model that allows it to reach higher accuracy levels (this distinguishes the *SPA* model from *gTTB*, in which the choice accuracy is quite limited; see below). We also illustrate how *SPA* and *WAV* decision strategies make distinct predictions regarding response times. Second, we present the results of a laboratory experiment where we evaluate these predictions, and in which we additionally collected participants’ confidence about their choices. To anticipate our results, our data supports the idea that participants deploy a compensatory strategy in a fast and automatic manner. Moreover, our results are not compatible with the generalized version of the *TTB* strategy (or *SPA* model), unlike what was found in the context of learning of binary cues (Bergert & Nosofsky, 2007). To reconcile the tension between compensatory and non-compensatory strategies, some authors have suggested that both strategies can be implemented within the same computational framework, of sequential sampling and accumulation of evidence (Lee & Cummins, 2004), or parallel processing of attributes (Glöckner, et al., 2014). We return to this in the discussion section.

## Simulation 1: Choice patterns and performance of *SPA* and *WAV* models

The goal of our first simulation is to confirm that the proposed *SPA* algorithm can mimic choice weights of the normative *WAV* solution.

### Method

Consider a multi-attribute decision task that requires selecting the best out of two job candidates A and B, based on their ratings (*A*_*i*_ and *B*_*i*_) on three job-related attributes. The three attributes do not have the same importance, and the importance of each attribute is prescribed by the experimenter. One example of this task is illustrated in Table 1.

**Table 1:**
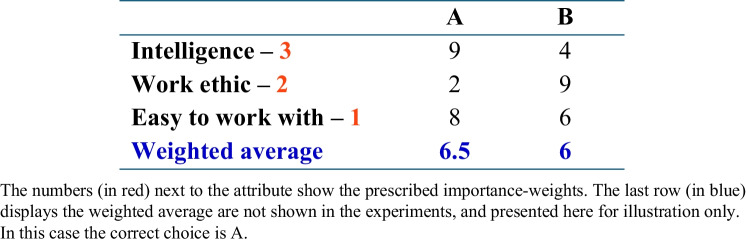
A typical stimulus from the job candidate task with three attributes

The normative strategy is to compute the weighted average (*WAV*) of the attribute values according to the importance of each attribute, and to pick the candidate with the highest weighted average value. It is convenient to consider using normalized weights, noted *w*_*i*_, where each *w*_*i*_ is proportional to the importance of attribute *i* and the sum of all *w*_*i*_ across attributes equals one. The decision variable for this strategy *DV*_*WAV*_ can be calculated as the weighted average of difference scores, as in Eq. 1, and the decision rule is then to select A if this decision variable is positive and B otherwise.

1 $${DV}_{wav}={\sum }_{i=1}^{3}{w}_{i}\left({A}_{i}-{B}_{i}\right)$$

**Fig. 1:**
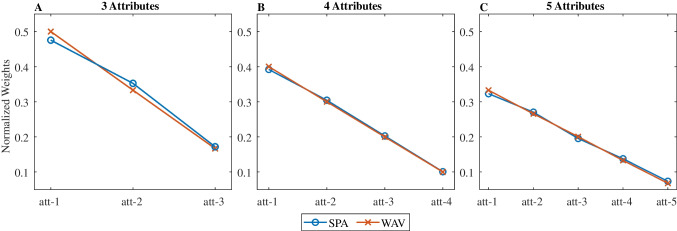
Average logistic decision weights (normalized) for the *SPA* and *WAV* models, for 3, 4 and 5 attributes choices. Red line with X symbols are the *WAV* weights, and blue-line with O symbols are the *SPA* weights. (Color figure online)

We introduce here an alternative strategy, which we call single probabilistic attribute (*SPA*), and which expands on Tversky’s *elimination by aspects* heuristic (Tversky, 1972; see also Bergert & Nosofsky, 2007; Nosofsky & Bergert, 2007). Under this strategy (which corresponds to the “one-reason decision-making: strategies, described by the heuristic program; Todd & Gigerenzer, 2000) the individual selects only one attribute (out of the three) and then chooses the candidate with the highest score on the basis of this attribute alone. The selection of this single attribute is probabilistic: for each attribute *i*, with probability *p*_*i*_, this attribute will be used for the decision. Importantly, if these probabilities *p*_*i*_ are set equal to the attribute normalized weights *w*_*i*_, then the *SPA* strategy is quite similar to a noisy version of the normative strategy (Eq. 1) if one only looks at choices. To formally show this, we simulated choices under the *WAV* and *SPA* strategies, on 8,100 randomly generated problems with the structure illustrated in Fig. 1.,^6^ ^7^ On each trial, two stimuli are defined by three attributes whose values are integers drawn uniformly and independently from the interval [1–9]. The prescribed weights of these attributes were always 3, 2, and 1 (from top to bottom in Table 1). For the *WAV* strategy, the noise level σ was chosen to obtain an 88% accuracy as in previous studies (see Fig. 1 in Brusovansky et al., 2018). For the *SPA* strategy, errors only came from the stochastic sampling of the attribute used for the decision. We generated 100 different simulated participants under both strategies, and for each simulated participant we regressed choices against the difference in attribute values. We then normalized the regression weights to sum to 1 and averaged these normalized weights across the 100 simulated participants. We repeated this simulation with problems based on four and five attributes (with weights, 4, 3, 2, 1 and 5, 4, 3, 2, 1, respectively).

### Results

Figure 1 shows the normalized weights for the *SPA* and *WAV* models (blue and red lines, respectively) and confirms that they do not distinguish between the two models: *SPA* can mimic the choice patterns of the normative solution.

In addition, we found that in trials where *TTB* and *WAV* produce opposite choices, *SPA* choices are equally similar to *WAV* and *TTB*.^8^ Thus, *SPA* is capable of approaching a compensatory strategy while being itself non-compensatory. However, when we examine decision accuracy (i.e., the probability of selecting the option with the highest *WAV*), we notice that in our simulations the accuracy of *SPA* was quite limited (Table 2).^9^ The reason for this is that *SPA* only relies on a single attribute (which is not always the most important one), and thus only provides limited evidence towards the correct choice, resulting in an upper bound on performance. Arguably, *SPA* (as well as *gTTB*) can be discarded for individuals who exhibit accuracy levels exceeding this bound. By contrast, *WAV* is only limited by decision noise, a free parameter that can accommodate any accuracy level.

**Table 2:** *SPA* model’s accuracy predictions for three levels of complexity (3, 4, & 5 attributes)

	3 attributes	4 attributes	5 attributes
*SPA* accuracy	73%	69%	67%

## Simulation 2: Introducing *SPA-D* to boost *SPA* accuracy

Because *SPA* is limited in terms of choice accuracy, in our second simulation we introduce a slight modification to the *SPA* algorithm, in order to achieve higher levels of performance.

### Methods

We designed a modified version of *SPA*, which importantly preserves the basic principle that the decision is determined by a single attribute at a time but imposes a constraint to ensure that this attribute provides enough evidence to guide the choice. Specifically, in the spirit of the lexicographic model (Tversky, 1969; see also priority heuristic, Brandstätter et al., 2006), we now assume that the selected attribute is used only if the evidence it provides (i.e., the difference between the two options on that attribute) exceeds a threshold level (*D*) in absolute value. If the evidence is below this threshold and if there are other attributes available, then a new attribute is sampled instead (without replacement). We call this new version the *SPA-D* model, where *D* is the threshold parameter. Note that when *D* = 0 this corresponds to the initial *SPA* model.

### Results

In Table 3, we show the average, and 95% confidence interval of the accuracy obtained under *SPA-D* with *D* ranging from 0 to 5. We find that accuracy increases from *D* = 0 to *D* = 3, where it reaches levels similar to that of actual participants.^10^ We also notice that the accuracy of *SPA-D* decreases after *D* = 4, presumably as the threshold on evidence strength becomes too high, such that more trials end with the choice being postponed to the last attribute (which is more likely the attribute with the lowest weight and least predictive of the correct choice). We thus focus on *D* = 3 in what follows.

**Table 3:** Choice accuracy of the *SPA-D* model

	*D* = 0	*D* = 1	*D* = 2	*D* = 3	*D* = 4	*D* = 5
3 attributes	.73 (.65,.79)	.76 (.69,.83)	.82 (.75,.88)	.83 (.77,.89)	.82 (.75,.87)	.78 (.71,.85)
4 attributes	.69 (.61,.76)	.72 (.65,.79)	.77 (.71,.84)	.80 (.74,.87)	.80 (.73,.86)	.76 (.69,.83)
5 attributes	.67 (.60,.75)	.70 (.63,.77)	.74 (.67,.81)	.77 (.71,.83)	.78 (.71,.84)	.76 (.69,.83)

The accuracy (mean and 95% confidence intervals) of choices made by the *SPA-D* model, in 10,000 simulations of 150 choice problems (as in Exp. 1). Simulations were conducted for all levels of *D* between 0 (i.e., initial *SPA* model) and 5, separately for the 3 levels of complexity (3, 4 & 5 attributes)

**Fig. 2:**
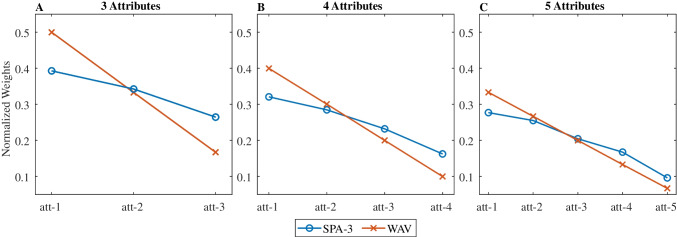
Average logistic weights (normalized) for the *SPA-3* and *WAV* models, for 3, 4, and 5 attributes choices. Red line with X symbols are the *WAV* weights, and blue line with O symbols are the *SPA-3* weights. (Color figure online)

The *SPA-3* decision weights (from logistic regression) are shown in Fig. 2 (in blue), and again mimic those of a compensatory strategy, despite *SPA-3* being a non-compensatory algorithm. The decision profile is even flatter than the normative weights (in red), because in some trials the choice is not determined by the attribute that is sampled first. We note that this flatter decision profile approaches the equal-weight heuristic (Dawes, 1979) deployed by some participants in empirical data (e.g., 8% of participants in Brusovansky et al., 2018).

Finally, we applied the classification procedure (see experiment 1, results section) to data generated by *SPA-3*, to determine whether *SPA-3* choices are more similar to *WAV* or *TTB*. The results favor *WAV* in 85%, 79% and 74%, for 3, 4, and 5 attributes, respectively.

In sum, participants whose decision weights show a compensatory pattern characteristic of *WAV* or of the equal-weight heuristic and whose accuracy does not exceed 89%, maybe potentially accounted by *SPA-3*. In the next section we turn to decision-time, as a complementary tool to contrast the models.

## Simulation 3: Response times of *SPA* and *WAV* models

Having shown that *SPA-D* can achieve reasonable levels of performance, we reasoned that *SPA* and *WAV* models may still be distinguished in terms of response times. Our next simulation in particular focused on how RTs depend on choice polarization^9^, here defined as the range, across attributes, of the differences between the two alternatives.

### Method

Table 4 illustrates an example of two trials with the same *WAV* difference but distinct levels of polarization^11^: in the right columns, the two choice options differ by − 2, + 2, or 0 on the different attributes (low polarization), whereas in the left columns they differ by − 6, + 6, or + 4 (high polarization). In both choice problems, the *WAV* difference is the same (.3), so the choice probability favors *A* according to both *WAV* and *SPA*. However, the two models make distinct predictions regarding how RT varies as a function of polarization.

**Table 4:** Two trials with the same difference in *WAV* between the options, but different polarization

	High polarization trial		Low polarization trial	
	A	B	A	B
**Attribute 1**	8	2	6	4
**Attribute 2**	2	8	4	6
**Attribute 3**	2	6	2	2
**WAV**	**5**	**4.7**	**4.7**	**4.4**

**Fig. 3:**
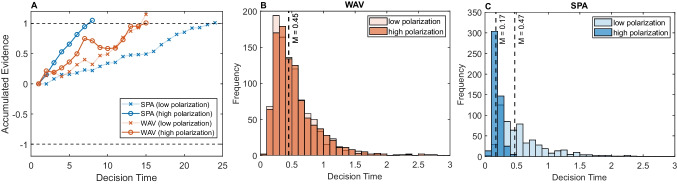
**A** Illustration of decision trajectory for two decisions stimuli, with the same *WAD* (Table 4) but different polarization (small, P-, and large P +), when the DDM drift is determined either by the *WAV* or *SPA* models. **B** RT distribution for *SPA* and *WAV* models under high and low polarization (N = 1000 simulations). The dashed black line is the median RT. (Color figure online)

To simulate RTs, we used a *DDM* approach (Glöckner & Betsch, 2008a, b; Lee & Usher, 2023; Ratcliff & McKoon, 2008) assuming that the decision-drift is based either on the *WAV* difference (Eq. 1) or for *SPA* on the difference in the sampled attribute (*A*_*i*_-*B*_*i*_)^12^; critically, for *SPA* in high polarization trials, this difference will typically be large irrespective of the specific attribute selected. By contrast, a low polarization *SPA* trial will not necessarily have a large difference on all attributes (see Table 4, for illustration). Therefore, under *SPA*, high polarization will induce a larger drift and faster responses than low polarization. By contrast, under *WAV*, RTs should only depend on *WAV* difference, but not on polarization per se. Figure 3A illustrates *DDM* trajectories and response times of *SPA* (blue) and *WAV* (red) models for the two-choice problem shown in Table 4, and Fig. 3B shows the full RT distribution for 1,000 simulations for these choice problems. As one can see, the median RTs of the two polarizations are the same under the *WAV* model but differ under *SPA*. While we do not develop a model of decision confidence here, based on existing models (e.g., Glöckner & Betsch, 2008a, b) we expect that the choice polarization will also correlate positively with confidence under *SPA* (at high polarization, one chooses based on a single attribute with a larger value difference).

To explore this effect of polarization on RTs more thoroughly, we then simulated RTs over a set of 8,100 choice stimuli, using the same stimulus set as before. In the present simulation, the decision boundaries and the noise level in the *DDM* were chosen so as to obtain ~ 88% accuracy on average.

### Results

**Fig. 4:**
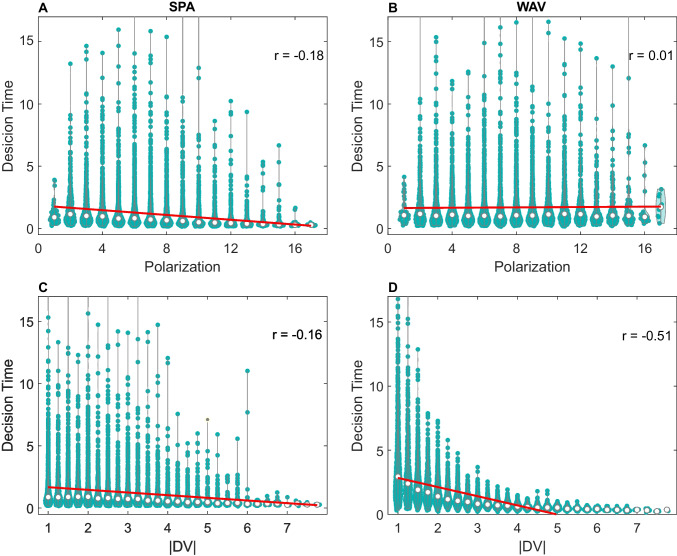
Upper panel: polarization–RT correlations under *SPA* (left) and *WAV* (right panel). *Y*-axis is the predicted RT by the model for 3 attribute choices. *X*-axis is polarization. Each dot is simulated RT for a specific trial. White dots are the median RT at each polarization level and the red line is the regression line. Bottom panel: $\left|DVwav\right|$ *RT* dependency in *SPA* and* WAV. Note.* while polarization affects RT in *SPA* but not in *WAV*, the opposite is true for $\left|DVwav\right|$*,* which affects RT in* WAV* more than in *SPA* (Eq. 1). (Color figure online)

Figure 4 (upper panels) illustrates how RTs simulated under the *SPA* and *WAV* models vary with polarization, across trials, for 3-attributes problems. Similar results are obtained with 4 and 5 attributes (see Supplementary Material Fig. A). In all cases, *WAV* and *SPA* models make distinct predictions regarding the correlation between polarization and RTs: while for *SPA* the RTs are negatively correlated with polarization (Fig. 4 top-left panel: *r* = −.18 for 3 attributes), this is not the case for *WAV* (top-right panel: *r* =.01 for 3 attributes). See Supplement for 4 and 5 attributes.

Unlike the *SPA* model, which shows RT dependency with polarization, the *WAV* model shows RT dependency with the difference in *WAV* between the options (Eq. 1). This is shown in Fig. 4 (bottom panels). Thus, the RT dependency on these two decision variables can dissociate between the models.

## Simulation 4: Response times of *SPA-D* model

In this fourth simulation, we show that the negative correlation predicted between polarization and RTs under *SPA* also holds for the *SPA-D* model.

### Methods

**Fig. 5:**
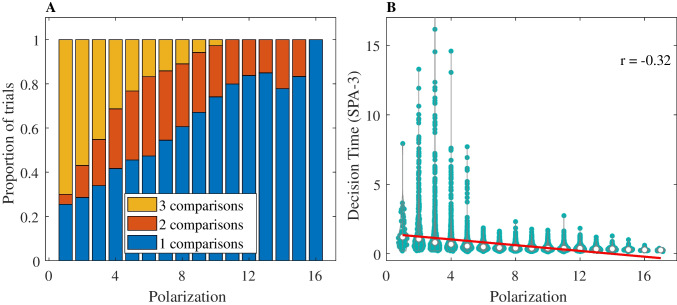
Left panel: Number of comparisons (1, 2, or 3) needed for the *SPA-3* model to reach a decision, within each polarization level. Right panel: polarization/RT correlation for the final comparison under *SPA-3*. Each dot is the simulated RT for a specific trial. White dots are the median RT of each polarization level. The red line is the regression line. (Color figure online)

In all *SPA-D* variants, the decision process is composed of two parts: a set of “failed comparisons” in which the attributes sampled do not provide evidence above the threshold, and a “final comparison” in which the attribute provides decisive evidence. From our previous simulation, we already know that under *SPA* the time associated with the “final comparison” is negatively correlated with polarization (Fig. 5). We can now examine how polarization affects the number of attributes that are sampled in the first part of the *SPA-D* process, in the same simulated dataset as used before.

### Results

Figure 5A (left panel) illustrates the effect of polarization on the number of attributes sampled before the decision is made, under the *SPA-3* model, for 3 attributes. Clearly, more attributes are sampled when polarization is lower. Figure 5B (right panel) illustrates the negative correlation between polarization and the decision time of the “final comparison” in *SPA-3*. Similar results are found for 4 and 5 attributes (see Supplementary Material).

In sum, in *SPA-D*, both the number of sampled attributes needed to reach a decision, and the duration of the final decisive comparison decreases with polarization. Thus, *SPA-D* necessarily predicts a negative correlation between polarization and RTs. The next section reports an experiment that evaluates this prediction, as well as two predictions of the *WAV* mechanism: The RT should decrease with the difference in the *WAV* between the options (Fig. 4, bottom panel) while the decision-confidence should increase with this variable (Glöckner & Betsch, 2008a, b).

## Laboratory experiment

The experiment follows Brusovansky et al. (2018), by presenting participants with speeded choices between pairs of job candidates. The number of attributes (task complexity) was 3, 4, or 5 (blocked). Our first aim was to replicate previous results by which participants are classified between *WAV* and *TTB*. Second, we aimed at evaluating the correlation between RTs and polarization, given that *SPA* and *SPA-D* models predict a negative correlation, whereas *WAV* model predicts no correlation.

### Method

#### Participants

A total of 54 participants (average age 23.3 years, *SD* = 5.4) were tested at the Centre d'Economie de la Sorbonne in Paris. The participants received compensation of 20€ in euros for their participation.

#### Materials and procedure

The participants were asked to select as fast and accurately as possible between two options, representing job candidates presented in a Table format (see Table 1, for illustration), and rated on 3, 4, or 5 relevant attributes, such as creativity, intelligence, and work ethic. The attributes were given specific importance weights that remained constant throughout the task. For instance, in the case of the 3-attribute condition, with the weights (3, 2, 1), “intelligence” held threefold higher importance than the “easy to work with” attribute. Attribute scores were drawn from a uniform distribution within the range [1, 9]. The sampling procedure was controlled, involving re-sampling in cases where the two alternatives shared the same weighted average.

Participants indicate which candidate was better by clicking on one of two response buttons located on either side of the screen. To calibrate participants' mouse positions, at the beginning of each trial, they also had to click on a fixed point positioned at the lower portion of the screen, equidistant between the positions of the alternative buttons. Decisions were constrained by time limits (3, 4, or 5 s for 3, 4, and 5 attributes problems, respectively). Slow response trials (exceeding the deadline) were excluded from subsequent analysis (1% on average). A brief training phase consisting of six trials preceded each complexity condition (3, 4, and 5 attributes). Feedback about accuracy (correct/incorrect) was delivered after each decision, as well as a warning in case of a slow response. After each decision, and prior to the feedback, participants also had to indicate their confidence. Decision confidence was included as an additional dependent variable that distinguishes heuristic (*TTB* or *SPA*) and *WAV* mechanisms (Glöckner & Betsch, 2008a, b). In addition, we wished to examine if participants who are classified as deploying a *TTB* strategy show sensitivity in their confidence choices between trials in which the choice they selected based on the most important attribute, aligns with the normative *DV*_WAV_, and those trials in which it does not.

Participants made 450 choices, with 150 choices for each complexity level (3, 4, and 5 attributes), organized in nine blocks of 50 trials each. A self-paced break was given after every set of 25 trials. All collected data was anonymized.

Open practice: The data and materials for all our experiments can be accessed online (https://osf.io/8qsnz/?view_only=bf512b7260884fa8827c76b44eaeed6c). None of the experiments were preregistered (Exp. 2 was carried out in parallel with Exp. 1, as part of another project).

#### Analyses

The main dependent variables in this experiment are choices, response times and confidence. Mouse trajectories were also recorded but not analyzed in detail.

For each participant, and for each complexity condition, we identified the set of trials where *WAV* and *TTB* make opposite choices and evaluated whether participants’ responses were aligned more with *TTB* or with *WAV* choices (see Brusovansky et al., 2018, for a similar approach). In addition, to test whether this classification as *TTB* or *WAV* was stable across complexity conditions, we used a permutation test. First, we calculated the actual number of participants showing consistent classifications across all three complexity levels. Second, we obtained a distribution of this consistency measure under the null hypothesis, by shuffling (5,000 times) the classifications across participants (within each complexity level) and applying the same calculation on each shuffled data. Finally, we located the percentile of the actual consistency with respect to the consistency distribution under the null hypothesis, which provides the *p*-value.

For each participant we also extracted decision-weights, and we carried out a quantitative model comparison on the choice data (we use BIC-fit measure) to compare the *WAV*, *TTB*, *gTTB*, and *SPA-D* choice models. Finally, for those participants who may be equally accounted for by *WAV* and (any) *SPA* version, we examined the correlation between polarization and RT to determine if they deploy a heuristic or a *WAV* mechanism. In all analyses of RT, we used median RT within each participant/condition, and then an average across participants.

### Results

#### Group accuracy and RT and confidence

Overall, participants performed the task quite well (overall accuracy: 0.84; average median RT: 1.5 s), replicating the results of Brusovansky et al. (2018). Separate within-subject ANOVAs conducted for accuracy, confidence, and for median RTs also indicated that participants were faster, more accurate, and more confident when they had to process stimuli with less attributes (accuracy: ${F}_{\left(\mathrm{2,106}\right)}=39.75,p<.001$; confidence: ${F}_{\left(\mathrm{2,106}\right)}=33.66,p<.001$, *p* <.001; response times: ${F}_{\left(\mathrm{2,106}\right)}=17.16,p<.001$). We then examined the correlations between the absolute value of $\left|DVwav\right|$ (normalized) with confidence and RT. We found that, as predicted by the *WAV* mechanism, an increase in $\left|DVwav\right|$ was associated with faster responses and higher confidence (average correlation between $\left|DVwav\right|$ and RT: *r* = −.27, *t*_(53)_ = − 25.17, *p* <.001; between $\left|DVwav\right|$ and confidence: *r* =.26, *t*_(53)_ = 14.66, *p* <.001). Thus, subjects’ choices were faster and more confident as $\left|DVwav\right|$ increase. All subsequent analyses were performed for each complexity separately.

#### Strategy classifications

**Fig. 6:**
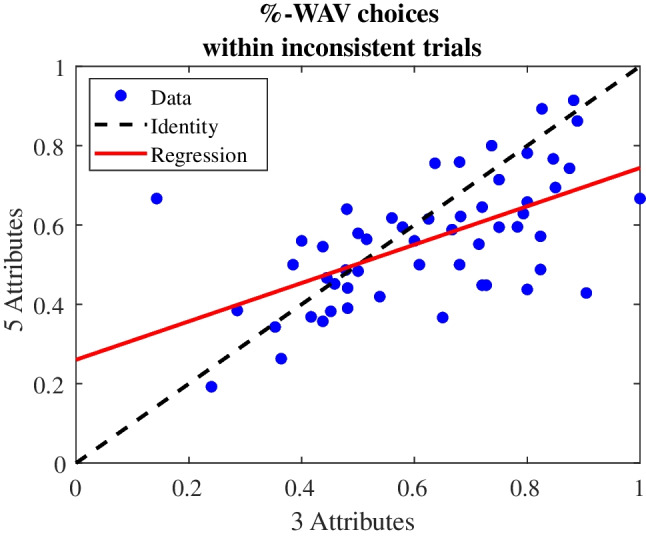
% *WAV* in 3 (*x*-axis) and 5 (*y*-axis) attributes conditions. Red line is the regression line Black dashed line is the identity line; blue dots are the individual participants. (Color figure online)

We classified participants according to whether their choices were more consistent with *WAV* or *TTB*, when the two strategies conflict (e.g., if the percentage, within conflict trials, of *WAV*-consistent choices exceed 50%, then the subject classified as *WAV*. Otherwise, the subject classified as *TTB*; see Bröder, 2010; Brusovansky et al., 2018, Supplement). Overall, about 69%, 69% and 63% of participants were classified as *WAV* in the 3, 4, and 5 attributes, respectively. These results replicate the classification results from Brusovansky et al. (2018). Furthermore, a stability test (see Methods) indicated that these classifications were stable over complexity conditions: participants classified as *TTB* or *WAV* in one complexity level tended to have the same classification in other complexity levels (*p* <.0001). Figure 6 shows a scatter plot of the percentage of *WAV* choices (%*WAV* within inconsistent trials) for the 3 and 5-attribute conditions, across participants. Each dot represents a participant %*WAV* in the 3-attribute condition (*x*-axis) and the 5-attribute condition (*y*-axis). As shown, the regression line (red) has a less steep slope than the identity line (black-dashed), indicating that although there is significant heterogeneity in the use of compensatory (*WAV*) and non-compensatory (*TTB*) strategies, the rate of *WAV* deployment decreases with increasing complexity. A repeated-measures ANOVA on the %*WAV* (within conflict trials) across three complexity levels yielded a significant effect for complexity. The mean proportion of *WAV* choices were.62,.57, and.56 for 3, 4, and 5 attributes, respectively (${F}_{\left(\mathrm{2,106}\right)}=5.34,p=.006$), indicating that as the choices become more complex (i.e., with a larger number of attributes), subjects rely less on a compensatory strategy (Pachur, 2022). The data also indicates a large degree of internal variability, indicated by the correlation of the two measures (%*WAV*−3 and %*WAV*−5) across participants (*r* =.59,* p* <.001).

**Fig. 7:**
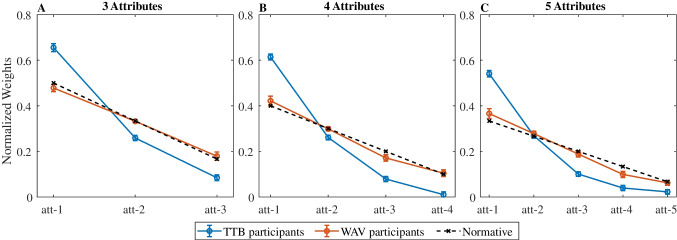
Decision weights by strategy. Mean decision weights (*y*-axis) for each attribute (*x*-axis), separately for participants classified as employing the *TTB* strategy (red) vs. *WAV* strategy (blue), along with the weights of the normative solution (black). Different panels correspond to different complexity levels (3, 4 or 5 attributes). Error bars indicate standard error of the mean across participants. (Color figure online)

Decision weights derived from logistic regression analyses confirmed that participants classified as *WAV* have close to normative weights, whereas those classified as *TTB* appear to overweight the first attribute and underweight the other attributes (see Fig. 7).

To evaluate the contention that the *TTB* strategy may allow for faster responses than *WAV* strategy, we also examined whether there is a difference in median RT between the participants that are classified as *TTB* vs. *WAV*. This difference (− 35, 75, and 84 ms for 3, 4, and 5 attributes, respectively) was not statistically significant in between-subjects ANOVA tests (${F}_{\left(\mathrm{1,53}\right)}<1$, *p* >.46 for all complexity levels), suggesting that adopting a *WAV* strategy was not costly in terms of response times, consistent with the *PCS* account (Glockner et al., 2008).

#### Model comparison on choice data

We fitted the *TTB*, *WAV*, *gTTB*, and *SPA-D* models to the choice data of all our participants, in order to determine if this data provides clear support to one of these models. A secondary aim of this model comparison was to determine how the *SPA-D* model compares with the *gTTB* model.^13^

In all the models we included a guessing parameter (see also Bergert & Nosofsky, 2007), which allows the production of probabilistic choices even in *TTB* (see Supplement for a full description of the choice models and of the fitting procedure). As the models vary in their number of parameters, we used BIC for model fits and model comparison. Before fitting the actual data, we first carried out a model recovery to ensure that the computational procedure can distinguish between data generated by the various models. To do so we generated 50 synthetic data sets for each model (by randomly sampling model parameter within the relevant parameter space; see Supplement for details) and we computed a confusion recovery matrix. As shown in Table 5, this shows a quite good model recovery. As expected, the higher confusion rates occur between* SPA-D* and *WAV*, and between *SPA-D* and *gTTB* (both ≤.16).

**Table 5:** Recovery matrix

Winning Model True Model	*SPA-D* (g, D)	*gTTB* (g, w_i_)	*TTB* (g)	*WAV* (g)
*SPA-D*	**80%**	0	4%	16%
*gTTB*	16%	**66%**	4%	14%
*TTB*	0	2%	**96%**	2%
*WAV*	0	0	2%	**98%**

For each model we specify its free parameters. *gTTB* has 4 parameters (3 weights and guessing), *SPA-D* has two parameters (*D, g*), while the other two models only one (g). We print the diagonal terms (which correspond to correct identifications) with bold

For each model we also carried out a parameter-recovery. The results (shown in Supplement) show high correlations (>.7) between the generated and the recovered parameters. Below we present a summary of the model fits separated along our classification strategy (*TTB* vs. *WAV*; see Supplement for the individual data). We start with the 3-attribute task (Table 6).

**Table 6:** Average BIC for *TTB* and *WAV* groups (3 attributes)

Fitted model Classification	*gTTB* BIC (4)	*SPA-D* BIC (2)	*WAV* BIC (1)	*TTB* BIC (1)	*N*
*TTB*	94.3 (23.6)	109.8 (17.1)	108.2 (21)	**92.4** (27.5)	17
*WAV*	129.8 (25.7)	120.1 (23.5)	**115.8** (32.2)	146 (24.3)	37
Total	118.7 (30)	116.9 (22.1)	**113.4** (29.2)	129.1 (35.5)	54

The numbers in parentheses, under the model’s name, are the number of parameters of each model. The numbers in parentheses, after the model’s average BIC, is the standard deviation across participants. The lowest BIC model within each row is shown in bold

**Fig. 8:**
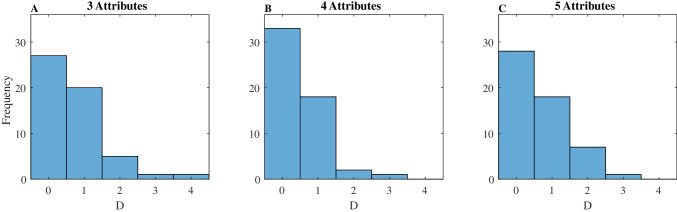
Distribution of the D parameter. From left to right: 3–5 attributes conditions

For the 3-atrribute task, across the whole sample *(N* = 54) we found that *WAV* was the best model overall, followed by *SPA-D*. We also found that *gTTB* outperformed *TTB*, replicating Bergert and Nosofsky (2007). However, in our sample, both the *SPA-D* and *WAV* models obtained a better fit than the *gTTB*. When examining the results for the two strategy classifications, separately, the picture becomes more interesting. For *TTB-*classified participants, while *gTTB* exceeds the performance of *SPA-D* and *WAV*, the best model is *TTB* (with only guessing); this is probably the outcome of *TTB* requiring fewer fit parameters. On the *WAV*-classified participants, the best performing models are *WAV* and *SPA-D.* The* SPA-D* model fit shows variability in the *D*-parameter (see Fig. 8), which allows it to maintain accuracy in the required range.^14^

The results in the 4-attribute task are similar (Table 7). In this case the across all participants, the *gTTB*, *SPA-D,* and *WAV* result in quite similar fits, which are all better than *TTB*. When looking at participants classified as *TTB*, again we find that *TTB* provides the best fit (followed by *gTTB*), while for the *WAV*-classified participants, we find that *WAV* and *SPA-D* provide the best fits.

**Table 7:** Average BIC for *TTB* and *WAV* groups (4 attributes)

Fitted model Classification	*gTTB* BIC (5)	*SPA-D* BIC (2)	*WAV* BIC (1)	*TTB* BIC (1)	*N*
*TTB*	114.8 (19.8)	131.8 (13.5)	138.2 (19.7)	**113.4** (20)	17
*WAV*	148.8 (27.2)	142 (22.1)	**140.1** (25.9)	163.7 (26.9)	37
Total	**138.1** (29.6)	138.8 (20.2)	139.5 (24)	147.8 (34.2)	54

The numbers in parentheses, under the model’s name, are the number of parameters of each model. The numbers in parentheses, after the model’s average BIC, is the standard deviation. The lowest BIC model within each row is shown in bold

Finally, for the 5-attribute task, we find (see Table 8) that across all participants the best fitting model is *WAV*, which is also the best fitting model for the *WAV* participants. For the *TTB*-classified participants, again the best fitting model is *TTB*.

**Table 8:** Average BIC for *TTB* and *WAV* groups (5 attributes)

Fitted model Classification	*gTTB* BIC (6)	*SPA-D* BIC (2)	*WAV* BIC (1)	*TTB* BIC (1)	*N*
*TTB*	131.5 (34.4)	148.1 (20.7)	148.2 (21.2)	**126.1** (29.6)	20
*WAV*	156.8 (26.4)	148.8 (20.8)	**144.7** (26.2)	168.7 (27.6)	34
Total	147.4 (31.8)	148.5 (20.6)	**146** (20.3)	153.1 (34.8)	54

The numbers in parentheses, under the model’s name, are the number of parameters of each model. The numbers in parentheses, after the model’s average BIC, is the standard deviation. The lowest BIC model within each row is shown in bold

These results show the following patterns. The *SPA-D* and *gTTB* models show quite similar model fits, with some advantage for *SPA-D* in *WAV*-classified participants, and advantage for *gTTB* in *TTB*-classified participants. However, for this group of (*TTB* classified) participants the original *TTB* (with guessing) is the best fitting model. For the *SPA-D* model, we find that the average *D*-parameter is significantly different from zero (see Fig. 8): *M* = .69 (*U* = 4.78, *p* <.0001),* M* = .46 (*U* = 4.34, *p* <.001) and *M* = .65 (*U* = 4.65, *p* <.0001) for 3, 4, and 5 attributes, respectively.

Focusing on the *WAV*-classified participants, we see that the BIC difference between the best two models (*WAV* and *SPA-D*) are quite small (~ 5), with *SD* > 20, indicating that, as expected, the difference in model fit between these models is not diagnostic for distinguishing individual participants. Therefore, in order to do so we turn to RT analysis.

## Evaluation of the SPA strategy

So far, the results in terms of overall performance, classification or decision-weights closely replicate those of Brusovansky et al. (2018), indicating that most participants seem capable of adopting a compensatory strategy in fast online decisions. Our simulations however show that a non-compensatory strategy such as *SPA*, which only attends to a single attribute, can produce decision weights close to normative. Can *SPA* (or more generally *SPA-D*) account for the data of the participants classified as *WAV* in our previous analysis?

To address this question, we first note that for nearly half of these participants (16 out of 37 for 3 attributes; 9 out of 37 for 4 attributes; 13 out of 34 for 5 attributes), the *SPA* model can be excluded based on accuracy alone: these participants exhibit significantly higher accuracy than predicted by any version of the *SPA-D* (i.e., exceeding the upper bound of the 95% confidence interval; see Table 3). This leaves more than half of participants (21 out of 37, 28 out of 37, and 21 out of 34, for 3, 4, and 5 attributes, respectively) for whom accuracy lies below.89,.87, and.84 for 3, 4, and 5 attributes, respectively, and is thus compatible with both *SPA* and *WAV* accounts.

**Fig. 9:**
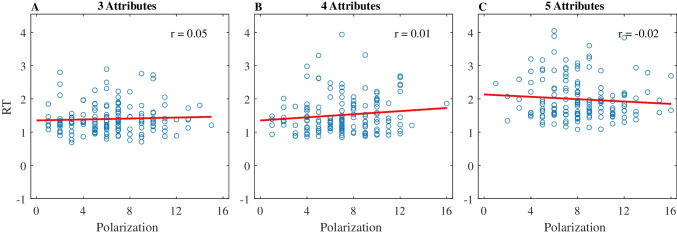
Polarization and RT correlation for representative subjects. *Y*-axis is the subject’s RT. *X*-axis is the polarization level. The red line is the regression line. **a** 3 attributes, **b** 4 attributes, and** c** 5 attributes. (Color figure online)

For this subset of participants (whom we label below *SPA*-candidates), we turned to RTs and more specifically how stimulus polarization affects RT to tease apart *SPA* and *WAV* accounts: As shown above, *SPA* predicts a negative correlation between RT and polarization (defined as the range in the choice evidence across attributes), whereas *WAV* predicts no correlation. (For completeness, we also conducted the same analyses on all *WAV* participants; see Table A in Supplementary Material.) Empirically, the average Pearson correlation in these participants was.05 (*SE* =.02),.01 (*SE* =.02) and −.02 (*SE* =.02) for 3, 4, and 5 attributes, respectively.^15^ Figure 9 illustrates a representative subject in each attribute condition. We computed Bayes factors in favor of the null correlation (${BF}_{01}$) and found an average of 8.48, 6.72, and 5.1 for the 3, 4, and 5 attribute conditions, respectively (only one subject had ${BF}_{01}$ < 1 in each of the 4 and 5 attributes conditions), providing substantial evidence favoring the absence of correlation, as predicted by the *WAV* model.

**Fig. 10:**
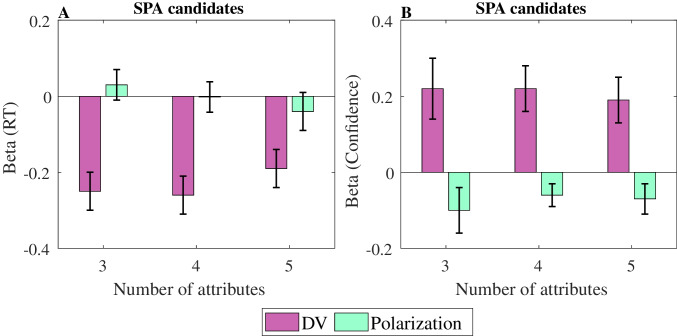
Regression beta coefficients of RT (left panel) and of confidence (right panel) with the $\left|DVwav\right|$ and with polarization. Error bars correspond to 95% confidence intervals. (Color figure online)

To further confirm this lack of correlation, we compared for each participant and each complexity level two linear regressions: one in which RTs were predicted solely by the difficulty level in each trial (as measured by the absolute value of *WAV*, $\left|DVwav\right|$), and another one that also included polarization as an additional predictor (Fig. 10, left panel), in addition to $\left|DVwav\right|$. We see that while the $\left|DVwav\right|$ clearly affects RT, the impact of the polarization is not consistent and the confidence intervals for its coefficient always include zero. A comparison of the two regression models on Bayesian information criterion (BIC; see Table 9) showed lower BIC for the first regression in each complexity level, thus favoring the hypothesis that polarization should not be included as a predictor of RTs. This was also true at the individual level for all participants (21 out of 21 in 3 attributes, 28 out of 28 in 4 attributes, and 18 out of 18 in 5 attributes). Across participants, a paired *t*-test indicated significantly lower BIC values for the regression without the polarization variable, at each complexity level (${t}_{\left(20\right)}=-12.53,{t}_{\left(27\right)}=-12.05,{t}_{\left(27\right)}=-10.12$; all *p* <.000001). In sum, our experimental data provides clear evidence against the negative correlation between polarization and choice RT predicted by *SPA*.

**Table 9:** BIC values of the regression models

	3 attributes	4 attributes	5 attributes
Without polarization	168.76 (8.18)	245.91 (11.47)	287.05 (15.64)
With polarization	172.52 (8.2)	249.6 (11.41)	291.61 (15.64)

Average BIC values across participants, in each complexity level, for a regression where RTs were predicted from difficulty or from difficulty and polarization. Numbers in parentheses are *SE*s

Finally, we carried out a similar regression with $\left|DVwav\right|$ and polarization as predictors for confidence responses, which provided converging evidence against *SPA*. Indeed, as shown in Fig. 10 (right panel), confidence not only increased with $\left|DVwav\right|$ but also significantly decreased with polarization, whereas under *SPA* one would expect a positive relation between confidence and polarization (due to faster and more confident responses for high polarization trials).

## Evaluation of the TTB strategy

**Fig. 11:**
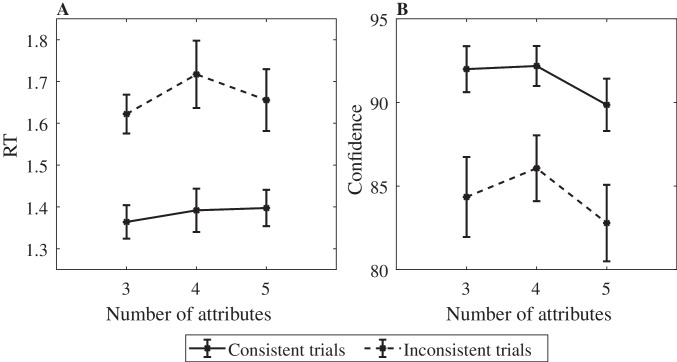
Confidence (right panel) and RT (left panel) as a function of consistency (between *TTB* and *WAV* choice-prediction). *Y*-axis is confidence (right panel)/RT (left panel). *X*-axis is the number of attributes (3–5). Dashed line = inconsistent trials; solid line = consistent trials. Error bars are 1* SE*

For *TTB* subjects, we examined whether RT and decision confidence differ between trials in which the *TTB* and *WAV* strategies lead to the same choice (no-conflict trials) and trials in which they lead to opposite choices (conflict trials). For RT, we found that conflict trials had longer RTs compared with no-conflict trials (${t}_{\left(16\right)}=4.07$, ${t}_{\left(16\right)}=5.3$ and ${t}_{\left(19\right)}=5.48$ for 3, 4 and 5 attributes conditions. All *p* < *.*001; see Fig. 11, left panel).

We also found that subjects classified as *TTB* show a higher confidence in no-conflict trials compared with conflict trials (${t}_{\left(16\right)}=7.04$, ${t}_{\left(16\right)}=7.27$ and ${t}_{\left(19\right)}=5.54$ for 3, 4 and 5 attributes conditions. All *p* < *.*0001; see Fig. 11, right panel).

However, we note that these last results could be due to conflict trials containing weaker evidence in the first attribute rather than the occurrence of conflict per se. Indeed, weak evidence in the first attribute would lead to slower and less confident responses in *TTB* participants (which mostly rely on this first attribute), but it would also make a conflict more likely, because this weak difference can more easily be overturned by the evidence in the remaining attributes. To address this issue, we predicted RT and confidence in separate regressions, based on the absolute value of the difference in the first attribute and conflict. We found that for both RT and confidence, the fixed effect of conflict was highly significant across all levels of complexity (all *p* <.0000001). These findings suggest that the evidence in the first attribute alone cannot fully explain the slower RTs and lower confidence ratings. These results indicate that even when a participant decides based on the most important attribute, conflict with other (less important) attributes is registered and affects decision confidence and RT.

### Experiment 2

To examine if the experimental results in Exp.1 are robust, in particular with regards to lack of a negative correlation between choice RT and polarization, we ran a replication experiment. The mean accuracy was .86 and.83 for 3 and 4 attributes-conditions, respectively and the average median-RT was 1.06 and 1.12 for 3 and 4 attributes, respectively. These results confirm that subjects are able to make relatively fast and accurate complex decisions. About 51% and 40% of the participants were classified as *WAV*, for 3 and 4 attribute conditions, respectively. The rest of the results (presented in the Supplement) confirmed the conclusions of Exp.1 regarding the absence of a negative correlation between decision-time and decision polarization. Note that this replication was carried out in a different country with a new sample of a larger size (*N* = 63; see Methods of Exp. 2 in Supplementary Materials). For transparency, we also wish to acknowledge that this experiment was part of a larger project, which additionally included a task on the perception of summary statistics as well as personality questionnaires, and which will be the topic of a separate publication.

## Discussion

In two experiments, we tested whether the data in a speeded multi-attribute decision task with prescribed importance weights are more consistent with the prediction of a compensatory *WAV* or of a heuristic non-compensatory *SPA* model that produces identical decision weights. While the *SPA* performance on the task is bounded (Table 2), it is possible to expand *SPA* into a series of heuristic variants that enhance its performance, by introducing a comparison threshold that must be exceeded for a comparison to be considered decisive. We labeled those *SPA* variants *SPA-D* and showed how performance depends on this threshold *D* (Table 3). This revealed that for some participants (about 63%), decision accuracy is compatible with *SPA-D*, and that decision weights considered as indicative of a compensatory strategy such as *WAV* could still be accounted via a heuristic *SPA* variant, which does not compute a weighted average. A quantitative model comparison based on choice data provided consistent results, showing that for *WAV*-classified participants (about 70%) the *WAV* and *SPA-D* models provided the best fits, but with small differences between them.

To further distinguish between *WAV* and the *SPA*-types models,^16^ we turned to decision-time. According to the simple *SPA* model, one expects a negative correlation between choice RT and choice polarization (Fig. 4, left panels), whereas according to the *WAV* model no correlation is expected (Fig. 4, right panel). In the *SPA-D* variants the same negative correlation is predicted, as the additional component in these models, the number of sampled attributes, also increases with choice polarization (Fig. 5A). The examination of the experimental data provided strong evidence against the *SPA* hypothesis and was more consistent with the *WAV* prediction (compare Figs. 4 and 8). These results were obtained in 3 blocks of trials that varied the number of choice attributes, from 3 to 5, respectively, and they were replicated in two different samples of participants. Our study thus supports the proposal that human participants have the capacity to carry out fast and compensatory decisions in multi-attribute choice with numerical values (Brusovansky et al., 2018), as they do in probabilistic inferences tasks with binary cues (Glöckner & Betsch, 2008a). These conclusions are consistent with those obtained by Glöckner and Betsch (2008a, 2012) who show that choice RT in multi-cue probabilistic inference tasks, decrease with what we labeled the $DVwav$, even to the degree that adding information to a decision problem can speed up RT, when this information increases the $DVwav$ (Glöckner & Betsch, 2012). 

### Comparing gTTB and SPA as probabilistic non-compensatory processes

Our findings, however, are opposite to the conclusions of Bergert and Nosofsky (2007), who have shown that a generalized version of the *TTB* heuristic (called *gTTB*) better accounts for participants’ subjective weights and response times in an inference learning task with binary cues. Specifically, they found that most participants give weight to a single attribute (see Bergert & Nosofsky, 2007, Table 7) and that response times do not depend on the evidence magnitude considered by the rational compensatory model (equivalent to *WAV*), supporting the idea that they use of a non-compensatory strategy. By contrast, as shown in Fig. 7, while a minority of our participants show a somewhat similar weight pattern that overweighs the first attribute (blue curve in Fig. 7), the majority appear to deploy decision weights that are close to the normative (prescribed) values (red curves in Fig. 7; see Supplementary Material, Tables A–C, for data on individual participants). In addition, our finding that RTs depend on DVwav (Fig. 9) contradicts the RT result in Bergert and Nosofsky (2007). To better understand this discrepancy between our results and this earlier study, we now examine in more details the differences between them.

At the theoretical level, our *SPA* model is very close to *gTTB* (Bergert & Nosofsky, 2007; Nosofsky & Bergert, 2007), which also assumed that subjects sample attributes probabilistically, rather than in a fixed order. To our understanding, *gTTB* is in fact equivalent to *SPA-0,* except for two aspects: It includes for each individual a guessing component, as well as the possibility for decision weights to differ from normative weights (the latter is motivated by the specific learning experimental paradigm deployed in their study^17^). First, we note that the guessing component leads to lower accuracy in *gTTB* than in *SPA-0*, and therefore plays against the possibility of *gTTB* accounting for our empirical data, since accuracy for *SPA-0* was already too low to account for most participants in our experiments: only 5 (out of 54 participants) in Experiment1 and 4 (out of 63 participants) in Experiment 2 satisfy this low accuracy bound, for the 3-attributes condition.^18^ Second, and critically, as *gTTB* remains a non-compensatory process, it still predicts a negative correlation between polarization and decision times (see Supplementary Materials Section II), and this negative correlation was ruled out in our empirical data. In other words, our empirical data is inconsistent with both *SPA* and *gTTB*, whereas it can be explained by participants adopting a fast compensatory strategy, as suggested by the *PCS* model (Glöckner & Betsch, 2008a, b). We believe that the reason for the different conclusions of these two studies is that our experimental paradigms differ in several key aspects, which we shall now examine in detail.

### Experimental factors favoring compensatory and non-compensatory strategies

It is important to note, however, that the experimental paradigm examined by us and by Bergert and Nosofsky are quite different, both with regard to the task and to the stimuli used. While we examined a preference task, in which the importance of the attributes was transparently provided, Bergert and Nosofsky examined a probabilistic inference task (how poisonous various insects are), based on learning the cue-validity from error feedback. Therefore, the two studies probe quite different processes: i) learning of inference rules vs. ii) applying the rules in a speeded decision.

Why may the learning component impact on the use of heuristics?^19^ In Bergert and Nosofsky (2007) the cue validity was not provided but had to be learned from error feedback, based on a training set where the *TTB* and the *compensatory *strategy were systematically aligned. Thus, it is possible that what participants pick after their training is just one cue that is diagnostic for the task. An inspection of decision weights in Bergert and Nosofsky (2007, Table 7) indicates that most participants indeed converged on a single cue (most often the cue with the highest validity). Obviously, as participants have never experienced conflict between reliance on that cue and relying on a weighted average, it is quite natural that they would generalize this inference rule to new (test) trials. Thus, the deployment of a single-attribute heuristic in the test may be the mere consequence of the learning stage. In our experimental paradigm, by contrast, the weight and the values of all attributes were transparently provided on every trial (and the weights were constant across trials), so no learning was required, only an application of the prescribed rule in a speeded decision.

In addition, the tasks also differ on the type of stimulus used (complex visual pictures of insects, vs. numerical values in table format), which may matter in several ways. First, whereas numbers in a table are easily compared with one another as they all have the same format, for visual pictures of insects, the observer has to extract a different type of visual information for each cue (i.e., the specific shape of the antennae, the size of the legs, the texture covering the body, etc.). Second, given the degree of expertise one has with visual numbers, it is possible that these stimuli can be attended holistically to some degree (Rosenbaum et al., 2021), whereas the pictorial (insect) stimuli may require a more sequential visual process, which is likely to make the search of evidence more demanding. Finally, attributes in Bergert and Nosofsky (2007) take binary values, and thus the difference between two images is also all-or-none for each attribute, whereas our stimuli are numbers drawn between 1 and 9, such that differences for each attribute are more graded. This may facilitate the understanding that one small difference in an important (high weight) attribute can be compensated by large differences in a more minor attribute, in particular when all the weights are known and when all the values are easy to compare. These different aspects may all contribute to facilitating fast compensatory processing in our study.

We thus believe that the difference between our studies indicates an important contextual dependency in multi-attribute/cue decisions. While a probabilistic non-compensatory heuristic was favored when the participants need to learn the importance of the cues from error feedback, a true compensatory mechanism is favored when the importance of the cues is known and easy to access, and one only needs to combine them in a fast decision. It is also important to clarify that while the majority of our participants deployed a fast compensatory strategy, there was also a significant fraction (about 30% in Exp. 1 and about half in Exp. 2), who did deploy a non-compensatory heuristic. Based on the ecological and bounded rationality theory, it is quite plausible that this fraction would increase in conditions that would require a costlier evidence search (e.g., from memory rather than from “givens”; Gigerenzer et al., 1999).

### Can non-compensatory and compensatory strategies be unified?

It has been proposed that non-compensatory heuristics (such as *TTB*) and compensatory strategies may be both accounted for by the same process, within a sequential sampling and evidence accumulation framework (Lee & Cummins, 2004). In this framework, participants sample the attributes in the order of their importance and integrate the evidence up to a decision criterion. Critically, adopting a very low decision criterion will lead to fast decisions based only on the first attribute, thus mimicking the *TTB* strategy, whereas adopting a very high decision criterion will require all attributes to be examined and accumulated before a decision can be made, thereby implementing a compensatory strategy. There are two simple RT predictions that may allow us to test this model. First, across participants we should expect faster responses for participants classified as *TTB* compared with those classified as *WAV*. This, however, was not the case in our data: *TTB* participants were not significantly faster than *WAV* participants overall (${F}_{\left(\mathrm{1,53}\right)}<1$; as also reported in Brusovansky et al., 2018). Second, we would predict that for trials in which *TTB* and *WAV* strategies favor opposite options, choices consistent with *TTB* should be faster, because they would be based on less evidence. We found this prediction to hold only in participants classified as *TTB* in Experiment 1 (i.e., not in Experiment 2, and not for *WAV*-classified participants; see summary of results in Supplementary analysis; Table B). This indicates that while *TTB* decisions may involve a sequential sampling of the attributes to a decision criterion, *WAV* decisions are unlikely to involve a sequential scan of attributes. We believe that instead, *WAV* decisions in our task are based on a holistic mechanism in which all information is gathered and processed in parallel. One such model may be the parallel constraint satisfaction model proposed by Glöckner and Betsch (2008b), in the context of multi-cue inference tasks.

### Theoretical implications and perspective for future studies

One of the most influential studies for the adaptive use of heuristics in multi-attribute decisions was conducted by Payne et al. (1988). Building on the symbolic cognition framework (Newell & Simon, 1972), these authors proposed to quantify mental effort using “elementary information processing steps” (*EIP*; e.g., read an alternative value on an attribute into short term memory, or compare two alternative on an attribute),^20^ and showed that non-compensatory heuristics can save mental effort with a minimal decline in choice accuracy, especially under time pressure. Paradoxically, at the time when this study was published, the symbolic framework was strongly challenged by the parallel distributed processing (*PDP*) framework (McClelland & Rumelhart, 1981), where computation can take place in parallel, rather as a sequence of symbolic computations. Since then, *PDP* models (a.k.a. neural networks) have arguably become the dominant framework in many aspects of cognition from language (McClelland & Rogers, 2003; McClelland & Rumelhart, 1981; Seidenberg & McClelland, 1989) to vision (Krizhevsky et al., 2012). In the domain of decision-making, the *PCS* model proposed by Glöckner and colleagues (2014), illustrates how such parallel processing in a neural network model can compute a weighted average over a complex stimulus, without requiring an extensive number of processing steps.

This suggests that one should re-consider the assumption that compensatory computations that estimate weighted averaging are necessarily effortful. Whereas Payne et al. (1988) and others have shown that participants switch to heuristic strategies under time pressure, Glöckner and Betsch (2008a) results suggest that such transitions are due to the Mouselab decision environment itself, where information acquisition is sequential, slow and costly (participants need to click on each attribute-alternative combination to reveal its hidden value). By contrast, Glöckner and Betsch showed that in a probabilistic inference task with binary cues where information is available in parallel, individuals can apply compensatory strategies in less than 1.5 s. Such a result was extended to multi-attribute decisions with numerical (rather than binary) values by Brusovansky et al. (2018). The present study provides further support for these decisions being based on fast *WAV* comparisons, as it rules out probabilistic types of heuristic models, which could mimic *WAV* selection to some extent. Nevertheless, it is important to qualify that this conclusion does not apply to all the participants. Below we consider a potential account of this variability.

Recently, it was proposed that a parallel integration of binary cues in multi-cue inference task is facilitated by a subitization processes (Jevons, 1871; Kaufman et al., 1949), which allows observers to rapidly, accurately and effortlessly estimate the number of similar binary symbols for up to 4 attributes (Pachur, 2022). As the subitization range is 1–4, this motivated Pachur (2022) to suggest that conditions with a low number of binary attributes will promote compensatory decision strategies. While the effectiveness of subitization will not apply to sets of numerical attributes that need to be averaged rather than counted, we speculate here that there is an alternative mechanism that may replace it: the ability to extract summary statistics from arrays of perceptual or numerical elements (Ariely, 2001; de Gardelle & Summerfield, 2011; Michael et al., 2014; Rosenbaum et al., 2021). Future studies should examinine how individual differences in the use of compensatory strategies may relate to the ability to process summary statistics over stimuli.

As discussed above, our data also shows remarkable individual differences in decision strategy (as also pointed out by Bröder, 2003, and by Lee & Cummins, 2004). Similar to previous studies (Brusovansky et al., 2018) we find that while about 2/3 of the participants deploy on a fast compensatory (*WAV*) choice mechanism, about 1/3 of the participants appear to rely on a *TTB* heuristic. This classification was stable across the number of attributes, but with a small increase in non-compensatory strategies when the number of attributes increased from 3 to 5 (see Pachur, 2022). While previous studies have focused on the variability that is induced by the decision variables and environment, we found that an even larger variability appears across participants, even within fixed experimental conditions. Whereas some studies have explored the potential association between such strategy selection and cognitive capacities (WM-span and intelligence; Bröder, 2003),^21^ future studies could aim at identifying the stability over time of the decision strategy classification, and whether this variability across participants relates to individual differences in personality traits.

## Conclusions

Prior research has focused on the idea that heuristics can approximate normative solutions to decision problems. Echoing with this idea, we find here that the *TTB* heuristic can achieve reasonable levels of performance and that the *SPA* heuristic exhibit decision weights that appear as compensatory. However, we also illustrate how examining RTs can provide decisive evidence to arbitrate between them. In a multi-attribute task where participants had to compare two options described by numerical attributes and prescribed weights, we found clear evidence that a majority of participants adopt a normative weighted averaging strategy rather than such heuristics. Future research may examine empirical situations where *TTB* is the dominant strategy, to evaluate whether the minority of participants exhibiting compensatory choices may in fact be explained by the *SPA* heuristic.

## Supplementary Information

Below is the link to the electronic supplementary material.

Supplementary file1 (DOCX 851 kb)

## Data Availability

Data and materials uploaded on OSF repository.

## Footnotes

1 The use of the Priority Heuristic was suggested to account best for the choices of participants, when the ratio between the expected values was less than half. The authors suggest that “people may first look at the three (four) reasons, and if no difference is markedly larger than the others, they apply the priority heuristic” (Brandstätter et al., 2006, p. 426).

2 In such tasks, options are associated with several probabilistic cues. For instance, a juice vendor is described as having received positive/negative recommendations from several testers (cues), and these testers are known to provide accurate estimates of the product’s quality with a given probability (which can be learned by experience or stated explicitly to the participant).

3 These types of decision strategies are also labelled as weighted additive (*WADD*) utility. Here we use the more specific term of weighted averaging (*WAV*), as we specifically probe this more specific mechanism, by providing normative (prescribed) importance weights.

4 The decision accuracy was negatively correlated with RT (controlling for task difficulty), suggesting that prolonged deliberation did not enhance but rather reduced accuracy, contrary to conventional assumptions.

5 Another class of models that may be relevant are the decision by sampling models (Stewart et al., 2006), in which the decision is mediated by a sequence of ordinal comparison between sampled alternatives. Our *SPA* model, however, does not sample alternatives (which are fixed), but attributes and, in this sense, is more similar to the selective-sampling model (Vanunu et al., 2020), which samples outcomes from each option.

6 Throughput this paper we use italics for model labels (*WAV, SPA, TTB*).

7 This choice-set was used in Experiment 1, following the same design as in a previous study (Brusovansky et al., 2018), and we relied on it, in our present study.

8 The likelihood to classify data generate by *SPA* as compensatory rather than *TTB*, is 45%, 50% and 52%, for 3, 4 and 5 attributes, respectively.

9 This bound on choice accuracy also applies to *gTTB*, which determines the choice in the same way as *SPA-0*, except from a guessing component that can only further reduce choice accuracy, depending on the value of the guess parameter.

10 Participants in Brusovansky et al. (2018) reached an average accuracy of 90%, 86%, and 84% on comparable choice sets (for 3, 4, and 5 attributes, respectively), and many participants had an accuracy level within the 95% confidence interval of *SPA-D* accuracy.

11 A related term for decision-polarization, previously used in multi-attribute decision research is *choice*-*disparity* (Lee & Holyoak, 2021). It is important to distinguish between this sense of attribute-disparity (the degree to which individual attributes “disagree” with one another as to which option is superior; Lee & Holyoak, 2021), and a phonetically similar term, attribute-dispersion, which measures the dispersion of attribute weights (Payne et al., 1988). Our polarization is similar to attribute-disparity but not to dispersion.

12 This is consistent with the distance effect in single digit comparison (Dehaene et al., 1990; Moyer & Landauer, 1967).

13 We thank an anonymous reviewer for this helpful suggestion.

14 In all complexity conditions about half of the participants show a D > 0 parameter in the *SPA-D* fit. For these participants the *SPA-D* model has a better fit than a *SPA-0* (which is equivalent to a version of *gTTB* with fixed normative weights).

15 These correlations remain non-negative even after the RTs are logarithmically transformed. Moreover, support for the null hypothesis (via Bayesian analysis) remains unchanged.

16 This includes the *gTTB* model, which is equivalent, in term of RT predictions, to *SPA-0* (as further elaborated below).

17 Bergert and Nosofsky (2007, p. 109) write: “In our view, it is implausible that every subject learns and uses the objective cue validities from experience with the decision domain. We think it is more likely that subjects occasionally make mistakes in their assignment of weights to cues (e.g., see Newell & Shanks, 2003). Calculating cue validity for each dimension requires a great deal of memory capacity and experience with the domain (for similar arguments, see, e.g., Newell, 2005). For this reason, we consider natural generalizations of both *RAT* and *TTB* that relax the assumption that optimal feature weights are always learned and used. In these generalized models, the feature weights are simply free parameters.”

18 If we take a more conservative criterion of satisfying the accuracy bound of *SPA*-0 in all three complexity conditions, we have only two (out of 54) participants in Experiment. 1, and two (out of 63) participants in Experiment 2, who could be candidates for a* gTTB* account, based on choice accuracy only.

19 We thank an anonymous reviewer for pressing us to clarify this point.

20 Additional examples of EIPs are: i) add the values of two attributes in STM, ii) weight one value by another (product); see Payne et al. (1998, p. 535).

21 In (Bröder, 2003) an expected correlation between WM-capacity and compensatory strategy selection was not found, however, intelligence (as measure by a type of Raven Matrix test) was found to correlate with participants’ ability to adapt their strategy to the character of the environment.

## Code availability

The experiment generating code is available on OSF repository.
