# Supplementary material for: Contrasting holistic-compensatory with probabilistic heuristic strategies in multi-attribute decisions
Source: Psychon Bull Rev. 2026 Feb 27;33(3):101. doi: 10.3758/s13423-025-02795-2 (PMC12948877; doi:10.3758/s13423-025-02795-2)
Supplement: Supplementary file 1 — Supplementary file1 (DOCX 851 kb) [file 13423_2025_2795_MOESM1_ESM.docx]

# Supplement

***I. Additional SPA model predictions***
Figure A shows the polarization/RT correlation under *SPA* model, for 4 and 5 attributes complexity (see figure 4). As for the 3 attributes complexity, a negative (zero) correlation is predicted for the *SPA* model (*WAV*).


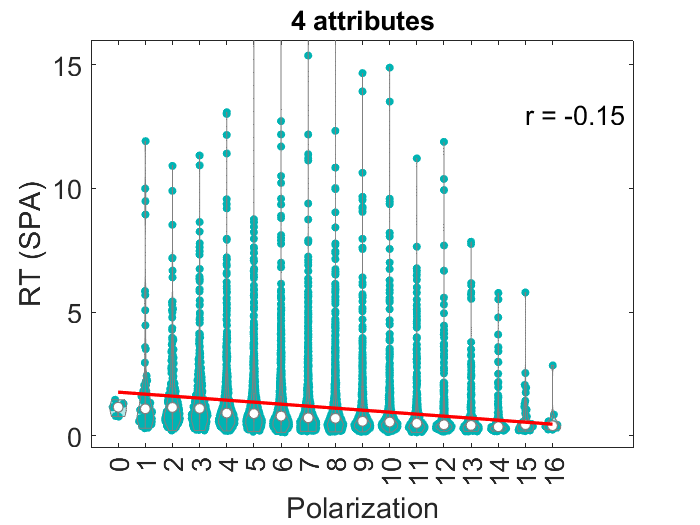

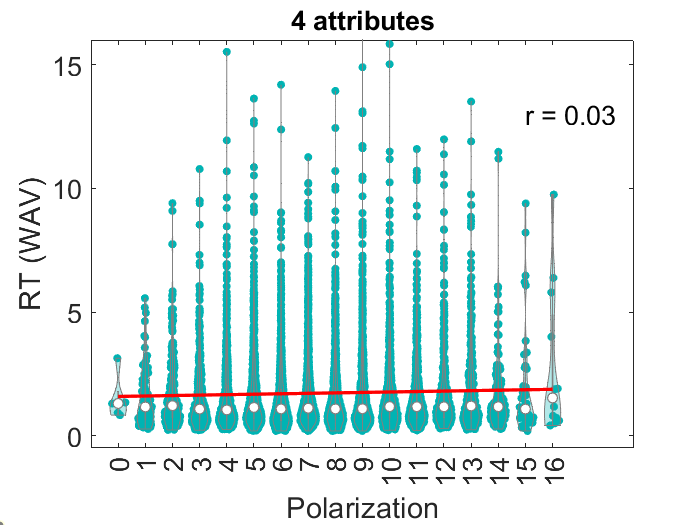


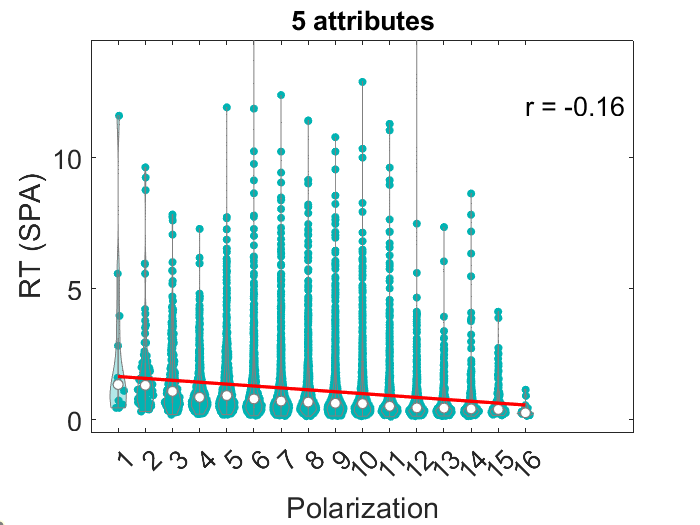

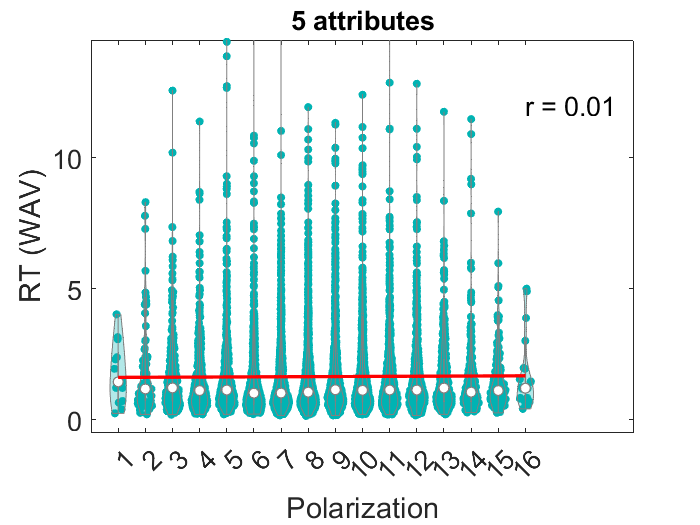


**Fig A.** *SPA and WAV Polarization/RT predicted correlations.* *Y-axis is the predicted RT by the model. X-axis is polarization. Each dot is simulated RT for a specific polarization choice. White dots are the median RT of each polarization level. The red line is the regression line. Left panel – SPA model, right panel – WAV model. Top panel – 4 attributes, bottom panel – 5 attributes.*

Figure B illustrates the effect of polarization on the number of attributes sampled before the decision is made and the negative correlation between polarization and the decision time of the “final comparison” under the *SPA-3* model (see figure 6).


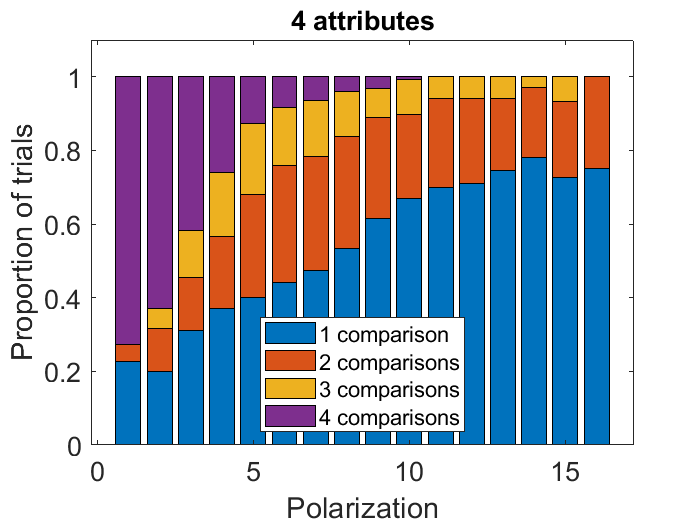

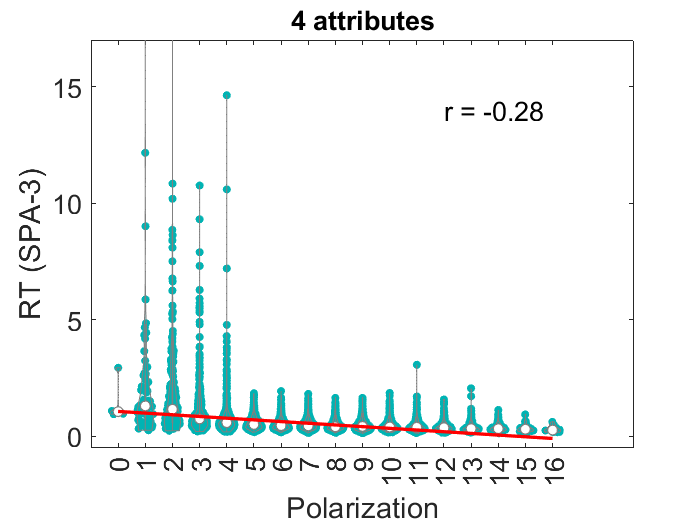


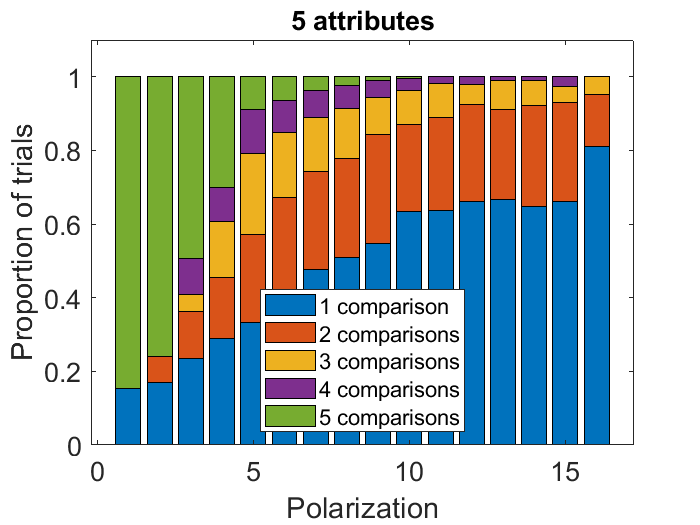

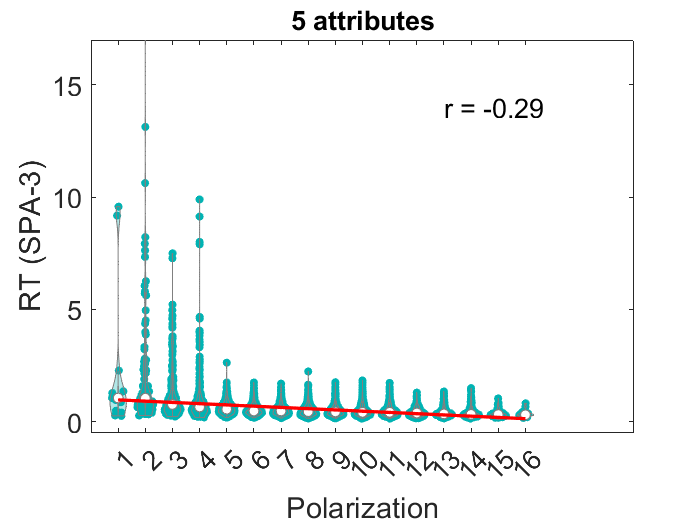


**Fig B.** *A) Left panel – SPA-3 model predicted proportion of trials for a given number of comparisons (1-k comparisons, k = 4,5) within each polarization level. B) Right panel – SPA-3 predicted polarization/RT (of the final comparison) correlations. Each dot is simulated RT for a specific polarization choice. White dots are the median RT of each polarization level. The red line is the regression line. Top panel – 4 attributes, bottom panel – 5 attributes.*

***II****.* ***gTTB* RT-predictions**

*gTTB* differs from *SPA* in two aspects:

1. The decision weights are free model parameters rather than normative prescribed values.
2. *gTTB* includes a guessing parameter, *g*, which controls the likelihood that a in a specific trial the participant will randomly guess, instead if applying any consideration of the choice stimuli.

We carried out simulations to check if these additional assumptions can affect the *SPA* prediction of a negative correlation between decision-time and decision polarization.


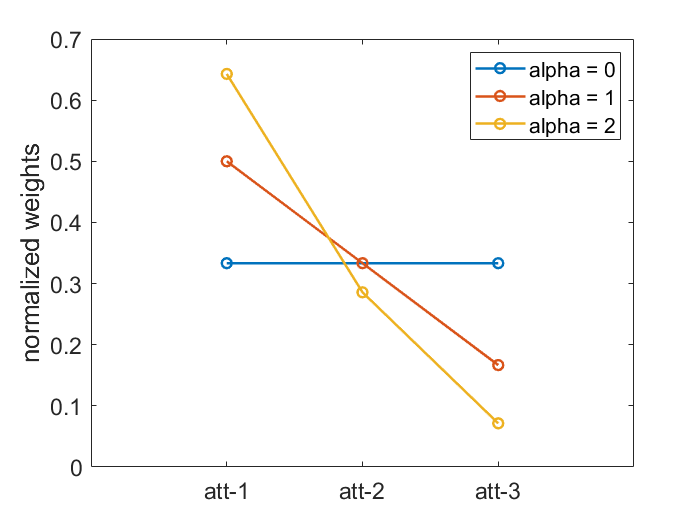
We first show that this prediction is insensitive to the shape of the weighting function. To do so we examined how the prediction is affected by a transformation of the normative weights (Brusovansky et al, 2018; Glöckner et al, 2014). One way to do this (see Brusovansky, et al, 2018) is to raise the normative weights, W_i_ to an exponent α, and then use the normalized W^α^*_i_*  as a measure of distorted weights. As shown in Fig C, for α = 1 this provides the normative weights, whereas for α = 0 it generates a distortion corresponding to the Equal-weight heuristic, and for α = 2 it generates a distortion in the direction of non-compensatory, *TTB*-like model (as is the case for *gTTB*).

**Fig C.** *Decision weights generated by the normalized W^α^_i_ (red corresponds to α = 1, blue to α = 0 and yellow to α = 2 – the TTB-like distortion). Note: the red line “alpha = 1” corresponds to the normative (prescribed) weights (normalized).*

We then repeated the Simulation-3 of the decision-time as a function of polarization, for an *SPA* model, which applies the distorted W^α^_i_ weights. This resulted in a negative Pearson correlation. As shown in figure D.


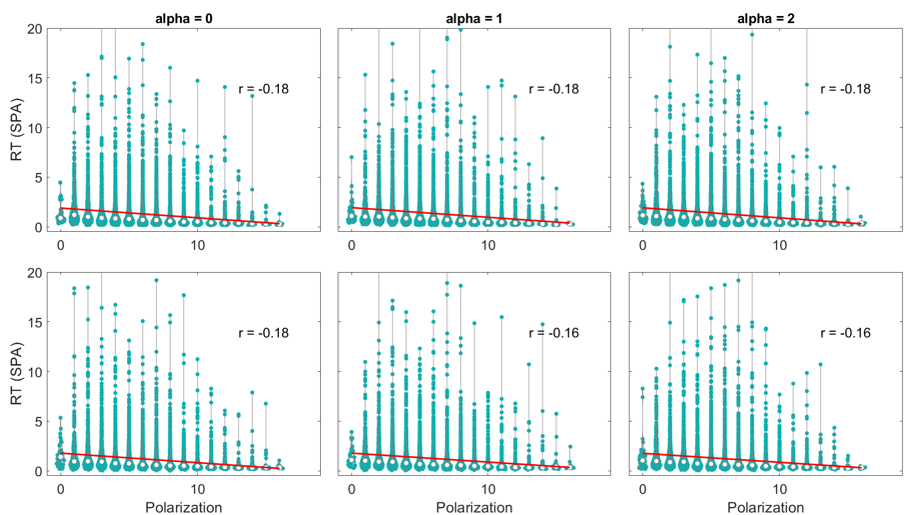


**Fig D.** *SPA Polarization–RT correlations under 3 α values (from left to right is α = 0,1 and 2) and with (bottom panels) or without (upper panels) guessing parameter (g).*

We then examined the impact of the guessing component. As Bergert and Nosofsky (2007) do not specify the value of the g-parameters and the RT distribution of the guess process, we use and estimate based on data; we set g = .1 (i.e., 10% of trials are guesses) and we assumed that guessing is relatively fast and independent of the choice stimuli. To implement this, we assumed that the RT guess distribution is Gaussian with mean selected at the 10% RT-quantile of the *SPA-0* (without a guess, i.e., Mean = .31 sec) and SD of .1. We used Bergert and Nosofsky formula for the probability of choosing A given a pair AB - $P\left( A|AB \right)=g/2+\left( 1-g \right)*{TTB}_{A}$ (Bergert & Nosofsky, 2007, p.114). With these assumptions we repeated the simulations above. The results are shown in the bottom panels of Fig C. Again, we see that *SPA* prediction of a negative correlation between RT and decision polarization is invariant to the guess and decision weight components.

*III.* ***Individual choice data in Exp.1***

Bolded cells are participants with accuracy lower than the *SPA* upper bound (table-3).

**Table A***Participants’ accuracy, strategy and choice-weights (logistic regression) for 3-attributes*

| **Participant** | **Strategy** | **Accuracy** | **w1** | **w2** | **w3** |
| --- | --- | --- | --- | --- | --- |
| 1 | WAV | 0.86 | 0.54 | 0.32 | 0.14 |
| 2 | TTB | 0.83 | 0.81 | 0.15 | 0.04 |
| 3 | WAV | 0.87 | 0.37 | 0.37 | 0.26 |
| **4** | **WAV** | **0.76** | **0.55** | **0.21** | **0.24** |
| 5 | WAV | 0.92 | 0.5 | 0.35 | 0.16 |
| 6 | TTB | 0.9 | 0.77 | 0.21 | -0.01 |
| **7** | **WAV** | **0.76** | **0.34** | **0.37** | **0.29** |
| 8 | TTB | 0.9 | 0.7 | 0.22 | 0.08 |
| 9 | TTB | 0.9 | 0.66 | 0.29 | 0.04 |
| 10 | WAV | 0.88 | 0.35 | 0.37 | 0.28 |
| 11 | WAV | 0.92 | 0.59 | 0.31 | 0.1 |
| 12 | WAV | 0.85 | 0.41 | 0.3 | 0.29 |
| 13 | WAV | 0.91 | 0.53 | 0.39 | 0.08 |
| 14 | WAV | 0.87 | 0.64 | 0.31 | 0.05 |
| 15 | WAV | 0.92 | 0.52 | 0.34 | 0.14 |
| 16 | WAV | 0.8 | 0.35 | 0.31 | 0.35 |
| 17 | WAV | 0.93 | 0.48 | 0.26 | 0.25 |
| 18 | TTB | 0.9 | 0.64 | 0.2 | 0.16 |
| 19 | TTB | 0.94 | 0.62 | 0.26 | 0.13 |
| 20 | WAV | 0.94 | 0.62 | 0.32 | 0.05 |
| 21 | WAV | 0.89 | 0.39 | 0.39 | 0.22 |
| 22 | WAV | 0.89 | 0.58 | 0.33 | 0.09 |
| 23 | WAV | 0.87 | 0.31 | 0.36 | 0.32 |
| 24 | TTB | 0.9 | 0.66 | 0.26 | 0.08 |
| 25 | TTB | 0.88 | 0.6 | 0.25 | 0.14 |
| 26 | WAV | 0.8 | 0.35 | 0.35 | 0.3 |
| **27** | **WAV** | **0.78** | **0.39** | **0.39** | **0.22** |
| 28 | WAV | 0.91 | 0.56 | 0.28 | 0.16 |
| 29 | TTB | 0.91 | 0.67 | 0.3 | 0.03 |
| 30 | WAV | 0.86 | 0.4 | 0.34 | 0.26 |
| 31 | WAV | 0.91 | 0.61 | 0.28 | 0.11 |
| 32 | TTB | 0.87 | 0.65 | 0.28 | 0.06 |
| 33 | WAV | 0.92 | 0.46 | 0.36 | 0.18 |
| **34** | **TTB** | **0.77** | **0.53** | **0.33** | **0.14** |
| 35 | WAV | 0.81 | 0.5 | 0.36 | 0.14 |
| 36 | WAV | 0.88 | 0.63 | 0.33 | 0.04 |
| **37** | **WAV** | **0.57** | **0.64** | **0.28** | **-0.08** |
| 38 | TTB | 0.88 | 0.63 | 0.28 | 0.1 |
| 39 | TTB | 0.92 | 0.75 | 0.23 | 0.02 |
| 40 | TTB | 0.85 | 0.6 | 0.35 | 0.05 |
| 41 | WAV | 0.92 | 0.46 | 0.31 | 0.23 |
| 42 | WAV | 0.84 | 0.48 | 0.25 | 0.27 |
| 43 | WAV | 0.95 | 0.5 | 0.4 | 0.1 |
| 44 | WAV | 0.88 | 0.49 | 0.39 | 0.11 |
| 45 | WAV | 0.89 | 0.46 | 0.28 | 0.26 |
| 46 | WAV | 0.88 | 0.52 | 0.36 | -0.12 |
| 47 | WAV | 0.92 | 0.58 | 0.35 | 0.07 |
| 48 | TTB | 0.86 | 0.59 | 0.28 | 0.13 |
| 49 | WAV | 0.86 | 0.34 | 0.35 | 0.31 |
| 50 | WAV | 0.82 | 0.36 | 0.32 | 0.32 |
| 51 | WAV | 0.89 | 0.41 | 0.35 | 0.24 |
| 52 | TTB | 0.91 | 0.68 | 0.22 | 0.1 |
| 53 | WAV | 0.91 | 0.48 | 0.33 | 0.19 |
| 54 | TTB | 0.86 | 0.58 | 0.27 | 0.15 |

**Table B**
*Participants’ accuracy, strategy and choice-weights (logistic regression) for 4-attributes*

| **Participant** | **Strategy** | **Accuracy** | **w1** | **w2** | **w3** | **w4** |
| --- | --- | --- | --- | --- | --- | --- |
| 1 | TTB | 0.82 | 0.57 | 0.24 | 0.1 | 0.09 |
| 2 | TTB | 0.83 | 0.62 | 0.22 | 0.1 | 0.06 |
| 3 | WAV | 0.81 | 0.3 | 0.25 | 0.33 | 0.13 |
| **4** | **WAV** | **0.61** | **0.28** | **0.36** | **0.14** | **0.22** |
| 5 | WAV | 0.84 | 0.45 | 0.25 | 0.22 | 0.07 |
| 6 | TTB | 0.88 | 0.57 | 0.27 | 0.14 | -0.02 |
| 7 | WAV | 0.8 | 0.45 | 0.31 | 0.15 | 0.1 |
| **8** | **TTB** | **0.76** | **0.66** | **0.22** | **0.04** | **-0.09** |
| 9 | WAV | 0.91 | 0.54 | 0.29 | 0.14 | 0.03 |
| 10 | WAV | 0.86 | 0.25 | 0.25 | 0.3 | 0.2 |
| 11 | WAV | 0.86 | 0.53 | 0.27 | 0.19 | 0.01 |
| 12 | WAV | 0.8 | 0.24 | 0.34 | 0.27 | 0.15 |
| 13 | TTB | 0.89 | 0.56 | 0.32 | 0.09 | 0.03 |
| 14 | WAV | 0.91 | 0.64 | 0.26 | 0.04 | 0.06 |
| 15 | WAV | 0.89 | 0.43 | 0.37 | 0.12 | 0.08 |
| 16 | WAV | 0.79 | 0.25 | 0.28 | 0.24 | 0.24 |
| 17 | WAV | 0.9 | 0.44 | 0.33 | 0.21 | 0.02 |
| 18 | TTB | 0.87 | 0.59 | 0.27 | 0.08 | 0.06 |
| 19 | TTB | 0.87 | 0.73 | 0.22 | -0.01 | 0.04 |
| 20 | WAV | 0.87 | 0.51 | 0.33 | 0.16 | 0 |
| 21 | WAV | 0.82 | 0.44 | 0.26 | 0.17 | 0.13 |
| 22 | WAV | 0.86 | 0.59 | 0.35 | 0.05 | -0.01 |
| 23 | WAV | 0.82 | 0.2 | 0.2 | 0.28 | 0.31 |
| 24 | TTB | 0.83 | 0.59 | 0.29 | 0.1 | -0.01 |
| 25 | WAV | 0.84 | 0.59 | 0.35 | 0.05 | -0.01 |
| 26 | WAV | 0.81 | 0.25 | 0.3 | 0.23 | 0.22 |
| **27** | **WAV** | **0.74** | **0.33** | **0.34** | **0.22** | **0.11** |
| 28 | TTB | 0.83 | 0.56 | 0.29 | 0.1 | -0.04 |
| 29 | TTB | 0.83 | 0.68 | 0.17 | 0.08 | 0.06 |
| 30 | WAV | 0.77 | 0.27 | 0.3 | 0.25 | 0.18 |
| 31 | TTB | 0.86 | 0.54 | 0.27 | 0.17 | 0.02 |
| 32 | WAV | 0.88 | 0.43 | 0.31 | 0.13 | 0.12 |
| 33 | WAV | 0.78 | 0.3 | 0.3 | 0.24 | 0.16 |
| **34** | **TTB** | **0.74** | **0.62** | **0.24** | **0.1** | **-0.04** |
| 35 | WAV | 0.85 | 0.52 | 0.32 | 0.1 | 0.05 |
| 36 | TTB | 0.81 | 0.54 | 0.33 | 0.1 | -0.03 |
| **37** | **WAV** | **0.7** | **0.45** | **0.3** | **0.06** | **0.18** |
| 38 | WAV | 0.87 | 0.54 | 0.34 | 0.11 | 0.01 |
| 39 | TTB | 0.83 | 0.66 | 0.24 | 0.04 | -0.07 |
| 40 | TTB | 0.88 | 0.67 | 0.27 | 0.03 | 0.03 |
| 41 | WAV | 0.86 | 0.46 | 0.29 | 0.21 | 0.03 |
| 42 | WAV | 0.8 | 0.36 | 0.24 | 0.27 | 0.13 |
| 43 | WAV | 0.9 | 0.5 | 0.38 | 0.07 | 0.05 |
| 44 | WAV | 0.86 | 0.5 | 0.37 | 0.12 | 0.01 |
| 45 | WAV | 0.81 | 0.35 | 0.21 | 0.25 | 0.19 |
| 46 | TTB | 0.81 | 0.61 | 0.32 | 0.01 | 0.06 |
| 47 | WAV | 0.86 | 0.52 | 0.37 | 0.09 | -0.02 |
| 48 | WAV | 0.87 | 0.56 | 0.33 | 0.08 | 0.04 |
| 49 | WAV | 0.83 | 0.25 | 0.26 | 0.27 | 0.22 |
| 50 | WAV | 0.78 | 0.26 | 0.28 | 0.28 | 0.18 |
| 51 | WAV | 0.81 | 0.48 | 0.36 | 0.09 | 0.07 |
| 52 | TTB | 0.79 | 0.66 | 0.25 | 0.07 | 0.02 |
| 53 | WAV | 0.89 | 0.5 | 0.28 | 0.18 | -0.04 |
| **54** | **WAV** | **0.73** | **0.64** | **0.13** | **0** | **0.23** |

**Table C**

*Participants’ accuracy, strategy and choice-weights (logistic regression) 5-attributes*

| **Participant** | **Strategy** | **Accuracy** | **w1** | **w2** | **w3** | **w4** | **w5** |
| --- | --- | --- | --- | --- | --- | --- | --- |
| 1 | WAV | 0.86 | 0.45 | 0.31 | 0.17 | 0.05 | 0.03 |
| 2 | TTB | 0.82 | 0.62 | 0.2 | 0.06 | 0.07 | 0.04 |
| 3 | WAV | 0.79 | 0.24 | 0.21 | 0.19 | 0.22 | 0.14 |
| **4** | **WAV** | **0.65** | **0.32** | **0.38** | **0.14** | **0.13** | **0.03** |
| **5** | **WAV** | **0.74** | **0.42** | **0.23** | **0.14** | **0.21** | **0.01** |
| 6 | WAV | 0.9 | 0.5 | 0.35 | 0.12 | 0.03 | 0 |
| 7 | WAV | 0.76 | 0.24 | 0.32 | 0.21 | 0.12 | 0.12 |
| 8 | WAV | 0.83 | 0.51 | 0.19 | 0.12 | 0.13 | 0.05 |
| 9 | WAV | 0.89 | 0.49 | 0.3 | 0.15 | 0.05 | -0.02 |
| 10 | WAV | 0.83 | 0.16 | 0.28 | 0.32 | 0.21 | 0.03 |
| 11 | WAV | 0.87 | 0.43 | 0.31 | 0.14 | 0.05 | 0.06 |
| 12 | WAV | 0.8 | 0.32 | 0.2 | 0.31 | 0.09 | 0.08 |
| 13 | WAV | 0.78 | 0.43 | 0.35 | 0.17 | -0.02 | 0.04 |
| 14 | WAV | 0.85 | 0.51 | 0.29 | 0.18 | -0.01 | -0.01 |
| 15 | WAV | 0.85 | 0.41 | 0.34 | 0.16 | 0.08 | 0 |
| **16** | **WAV** | **0.68** | **0.3** | **0.17** | **0.16** | **0.08** | **0.29** |
| 17 | WAV | 0.88 | 0.39 | 0.27 | 0.19 | 0.1 | 0.05 |
| 18 | TTB | 0.81 | 0.52 | 0.32 | 0.07 | -0.04 | 0.06 |
| 19 | TTB | 0.82 | 0.61 | 0.27 | 0.08 | 0.02 | 0.02 |
| 20 | WAV | 0.88 | 0.46 | 0.27 | 0.23 | -0.02 | 0.02 |
| 21 | WAV | 0.83 | 0.39 | 0.36 | 0.2 | 0.02 | 0.03 |
| 22 | WAV | 0.86 | 0.41 | 0.3 | 0.17 | 0.06 | 0.05 |
| 23 | WAV | 0.76 | 0.08 | 0.23 | 0.27 | 0.28 | 0.15 |
| 24 | TTB | 0.87 | 0.56 | 0.21 | 0.12 | 0.05 | 0.06 |
| 25 | WAV | 0.85 | 0.42 | 0.31 | 0.2 | 0.01 | 0.05 |
| 26 | WAV | 0.79 | 0.27 | 0.26 | 0.16 | 0.18 | 0.13 |
| **27** | **WAV** | **0.75** | **0.35** | **0.41** | **0.07** | **0.15** | **0.02** |
| 28 | TTB | 0.87 | 0.55 | 0.28 | 0.1 | 0.04 | -0.03 |
| 29 | TTB | 0.86 | 0.6 | 0.21 | 0.17 | 0 | 0.01 |
| 30 | WAV | 0.84 | 0.22 | 0.31 | 0.27 | 0.13 | 0.07 |
| 31 | WAV | 0.89 | 0.51 | 0.27 | 0.07 | 0.07 | 0.07 |
| 32 | TTB | 0.79 | 0.49 | 0.26 | 0.09 | 0.14 | 0.01 |
| 33 | WAV | 0.78 | 0.2 | 0.21 | 0.35 | 0.18 | 0.07 |
| 34 | TTB | 0.81 | 0.52 | 0.25 | 0.13 | 0.03 | 0.07 |
| 35 | TTB | 0.8 | 0.48 | 0.3 | 0.1 | 0.04 | 0.08 |
| 36 | WAV | 0.81 | 0.42 | 0.35 | 0.2 | -0.01 | -0.02 |
| **37** | **TTB** | **0.67** | **0.49** | **0.17** | **0.12** | **0.17** | **0.06** |
| 38 | TTB | 0.85 | 0.5 | 0.27 | 0.17 | 0.02 | -0.03 |
| 39 | TTB | 0.77 | 0.59 | 0.29 | 0.08 | -0.03 | -0.01 |
| 40 | TTB | 0.81 | 0.6 | 0.24 | 0.06 | 0.05 | 0.04 |
| 41 | WAV | 0.89 | 0.43 | 0.24 | 0.2 | 0.11 | 0.02 |
| 42 | WAV | 0.81 | 0.29 | 0.17 | 0.24 | 0.23 | 0.07 |
| 43 | TTB | 0.85 | 0.54 | 0.35 | 0.04 | 0.06 | -0.02 |
| 44 | TTB | 0.85 | 0.61 | 0.29 | 0.02 | 0.04 | -0.04 |
| 45 | TTB | 0.8 | 0.37 | 0.29 | 0.16 | 0.08 | 0.1 |
| 46 | WAV | 0.79 | 0.48 | 0.32 | 0.06 | 0.03 | 0.11 |
| 47 | WAV | 0.88 | 0.53 | 0.29 | 0.15 | 0.02 | 0 |
| 48 | TTB | 0.82 | 0.49 | 0.25 | 0.14 | 0.06 | -0.06 |
| 49 | WAV | 0.79 | 0.19 | 0.21 | 0.22 | 0.21 | 0.17 |
| 50 | WAV | 0.76 | 0.17 | 0.28 | 0.34 | 0.12 | 0.09 |
| 51 | TTB | 0.8 | 0.57 | 0.35 | 0.06 | 0 | 0.01 |
| 52 | TTB | 0.86 | 0.59 | 0.28 | 0.1 | 0.01 | 0.02 |
| 53 | TTB | 0.85 | 0.49 | 0.3 | 0.13 | -0.03 | 0.05 |
| 54 | WAV | 0.77 | 0.52 | 0.15 | 0.14 | 0.07 | 0.12 |

*IV.* ***Additional data analysis for Exp. 1***

As for the *SPA* candidates’ group (see table 5) we computed the BIC of the regressions with and without the polarization variable. Table-D summarizes the results of this analysis:

**Table D***BIC* *values of the regression models (all* WAV *participants)*

| **5 attributes** | **4 attributes** | **3 attributes** |  |
| --- | --- | --- | --- |
| 287.45 (1.92) | 245.97 (1.5) | 177.93 (0.96) | **Without polarization** |
| 291.28 (1.92) | 249.71 (1.5) | 180.98 (0.95) | **With polarization** |

In addition, we computed the Bayes factors in favor of the null correlation (
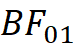
) for all WAV group and found 8.73, 6.92, and 5.17 for the 3, 4, and 5 attribute conditions, respectively.

The table below (Table-E) summarizes the results of the analysis testing the predictions of the Lee & Cummins (2004) unified model:

| **TTB group** | | **WAV group** | |  |
| --- | --- | --- | --- | --- |
| **Choices consistent with WAV** | **Choices consistent with TTB** | **Choices consistent with WAV** | **Choices consistent with TTB** |  |
| 1.85s | 1.48s | 1.7s | 1.69s | **3 attributes** |
| 1.95s | 1.55s | 1.77s | 1.71s | **4 attributes** |
| 1.96s | 1.55s | 1.89s | 1.76s | **5 attributes** |

**Table E***Average median-RT for trials consistent with TTB and WAV (separately for each group)*

The numbers in the table are the average median RT across subjects for trials in which subjects choose according to *TTB* versus trials in which they chose according to *WAV*. These trials were selected based on differing choice predictions between *TTB* and *WAV*. One subject was excluded from the analysis due to insufficient trials, as he/she did not choose according to *TTB* in any of the relevant trials. The difference in average RTs is significant for the TTB group across all levels of complexity (
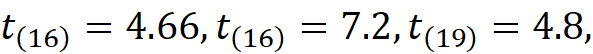
all p < .001), but not for the WAV group (
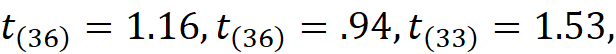
 all p > .14).

*Confidence data:*

We also calculated the correlation between polarization and confidence, controlling for
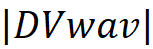
:

**Table F**
*Correlation coefficients across participants*

| **5 attributes** | **4 attributes** | **3 attributes** |  |
| --- | --- | --- | --- |
| -.08 (.01) ***** | -.06 (.01) ***** | -.11 (.02) ***** | **WAV-participants** |
| -.08 (.03) ***** | -.08 (.03) ***** | -.13 (.02) ***** | **TTB-participants** |

*Note:* *numbers in parentheses indicate 1 standard error;* ******* *denotes significant correlation.*

For the *TTB* group, subjects were less confident with higher polarization. For the WAV group, polarization had a smaller effect on confidence.

*V. Experiment 2*

**Participants**

To decide on the number of participants we conducted a power analysis on the difference in BIC values of the two regressions in Table-5. As the analysis indicated that 5 participants are enough to obtain an 80% power at .05 significance level, we decided to replicate the experiment with an equal number of Subjects (54). Considering possible cancellations, we invited 65 participants to participate, out of which 63 were tested. The participants were recruited via the SONA system and their average age was 24.27 (*SD* = 5.3). Participants could choose to receive course credit or 30 NIS (about 7.5€) payment. The experiment took place in Tel-Aviv University labs and was approved by the ethical committee of the Social Science board at Tel-Aviv University. Participants provided written consent for their participation.

**Method**

As in Experiment 1, except we used only the 3 and 4 attribute complexity levels, with 100 stimuli for each level. Participants received a self-paced break every 25 trials.

**Results**

First, we classified our subjects to WAV or TTB users with the same procedure carried out in experiment 1 (Brusovansky et al., 2018). As in experiment 1, we calculated the correlation between stimulus polarization (defined as the range in the stimulus evidence across attributes) and response times. The analysis conducted only for subjects classified as WAV, and with mean accuracy less than .89 and .87 for 3 and 4 attributes conditions, respectively (see table 3). The average Pearson correlation was .002 (*SE* = .03) and .005 (*SE* = .02) for 3 and 4 attributes, respectively. We calculated the Bayes factor (
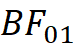
) of the polarization/RT correlation, as we did for experiment 1 – we obtained an average
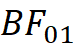
 of 5.23 and 4.96 for the 3 and 4 attributes, respectively (within the 3 attributes condition, only 2 subjects obtained
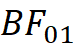
<1) – providing substantial evidence favoring the absence of correlation, as predicted by the *WAV* model, replicating the results of experiment 1. In addition, we calculated for each subject the BIC of two regressions – one with difficulty and polarization as predictor and the other with only difficulty (no polarization). Table-G summarizes the results of this analysis:

**Table G***BIC values of the regression models (experiment 2)*

| **4 attributes** | **3 attributes** |  |
| --- | --- | --- |
| 126.98 (16.15) | 81.2 (19.1) | **Without polarization** |
| 130.53 (16.16) | 84.1 (19.1) | **With polarization** |

The BICs (Table-G) were lower for the first regression for each complexity level, thus favoring the hypothesis that polarization should not be included as a predictor of RTs. This was also true at the individual level for all participants (17 out of 17 in 3 attributes and 18 out of 18 in 4 attributes), and a paired t-test also indicated significant difference in the BICs at the group level, with lower BIC on average for the regression without the polarization variable, at each complexity level (
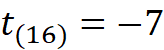
;
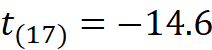
 all *p* < .0001). The table below (Table-H) summarizes the results of the analysis testing the predictions proposed by Lee & Cummins (2004) unified model:

**Table H***Average median-RT for trials consistent with TTB and WAV (separately for each group)*

| **TTB group** | | **WAV group** | |  |
| --- | --- | --- | --- | --- |
| **Choices consistent with WAV** | **Choices consistent with TTB** | **Choices consistent with WAV** | **Choices consistent with TTB** |  |
| 1.42s | 1.46s | 1.33s | 1.36s | **3 attributes** |
| 1.08s | 1.07s | 1.14s | 1.15s | **4 attributes** |

The numbers in the table are the average median RT across subjects for trials in which subjects choose according to *TTB* versus trials in which they choose according to *WAV*. These trials were selected based on differing choice predictions between *TTB* and *WAV*. 2 subjects were excluded from the analysis due to insufficient trials, as they did not choose according to *TTB/WAV* in any of the relevant trials. The difference in average RTs is not significant for both groups of subjects (all *p >= .4)*. In summary, for the WAV group, our experimental data does not support the negative correlation between polarization and choice-RT predicted under *SPA* (and *TTB*), thus supporting the *WAV* model. In contrast, for the TTB group, our data supports the negative correlation between polarization and choice-RT thus supporting the *TTB* model. Additionally, the data of experiment 2 does not support the unified model (Lee & Cummins, 2004). Overall, these results replicate the results of Exp. 1.

*V.* ***Model fitting of choice data***

50 synthetic subjects with 1500 synthetic trials (choosing between 2 alternatives with 3 attributes) were fitted for each model. We used fmincon (with MATLAB R2024b) for the optimizing routine with 15 different starting points. The negative Log likelihood (nLL) function is:

$$nLL= \sum_{i=1}^{N} -log({choice}_{i}*p_{i}+\left( 1-{choice}_{i} \right)*\left( 1-p_{i} \right))$$

(compare to equation B2 in appendix B in Bergert & Nosovsky, 2007).
N is the number of trials; ${choice}_{i}$ is the choice in trial *i* (equal to 1 if A is chosen and 0 for B); $p_{i}=\frac{g}{2}+\left( 1-g \right)*{pA}_{i}$ . *g* is the guessing parameter (Bergert & Nosofsky, 2007); *pA_i_* is the probability of choosing A given a choice problem (AB) in trial *i* (we can write it as ${p(A|AB}_{i})$). It is different for each model (see in each model section).

In all figures, the black dashed line is the Identity line.

***WAV (g) –***

This model is the deterministic *WAV* model; that is, the decision is made based on the weighted average difference. For compatibility with the other models (and the work done by Bergert & Nosofsky, 2007) we added the guessing parameter, g, to the model. Thus, P(A|AB) = 1 if $\sum_{i=1}^{k} w_{i}*(a_{i}-b_{i})$ > 0; otherwise, 0 (if the weighted average difference is 0, then P(A|AB) = .5). We used the same nLL function as with the other models; within the nLL formula, $p_{i}=\frac{g}{2}+\left( 1-g \right)*{pA}_{i}$ is the same as before, but in this model *pA_i_* is equal to 1 (for A) or 0 (for B).


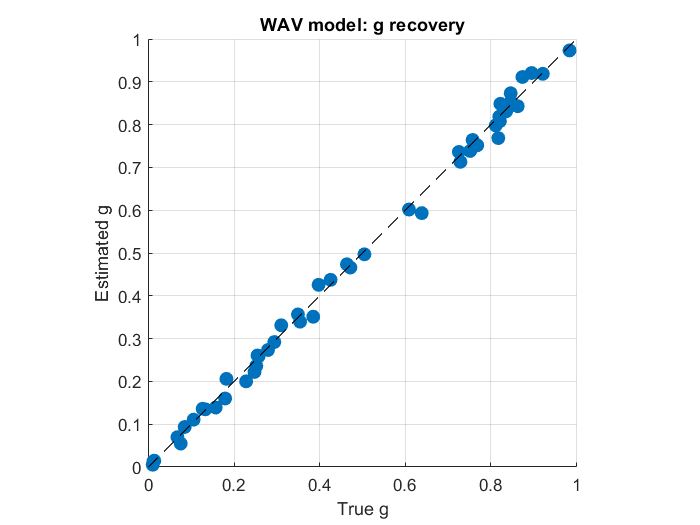
**Parameters recovery:**

The correlation between estimated g and true g – .99

***spaD (g and d) –***

$${pA}_{i}=\sum_{j=1}^{R} P\left( {order}_{j} \right)*I_{j}$$

P(order_j_) calculated the same as in equations 6-7 in Bergert and Nosovsky (2007), with the addition of the *d* parameter (i.e., an order favoring A is an order that leads the model to choose A, only if the first differentiating attribute difference exceed *d.* In *gTTB* d is fixed and equal to zero). R is all possible orders (permutations. For example, if there are 3 attributes, R = 3! = 6). *I* is an indicator of the order choice – if order_j_ favors alternative A, then *I_j_ =* 1, otherwise 0. As in *WAV* model, we are not fitting w_i_.


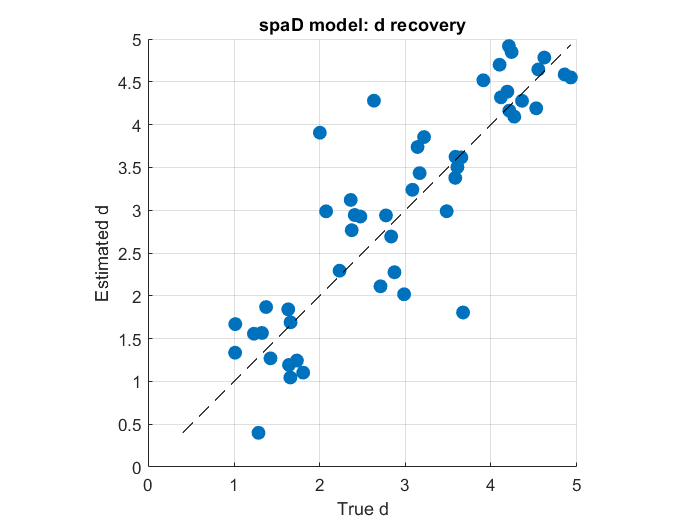
**Parameters recovery:**


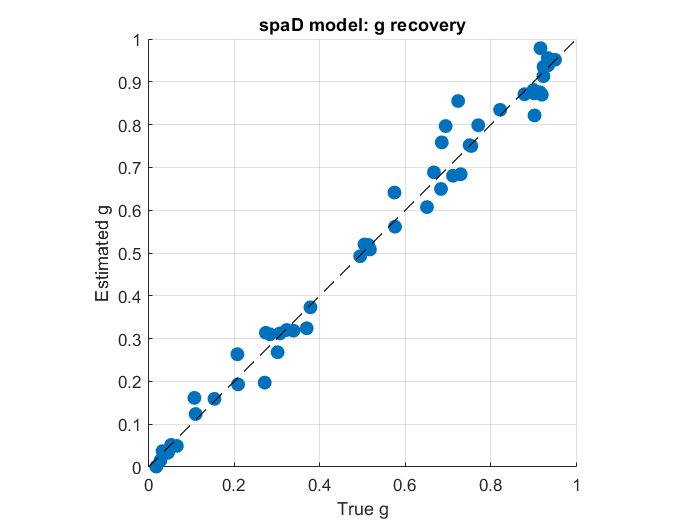


The correlation between estimated g and true g – .94

The correlation between estimated d and true d – .74

***gTTB (g and w_i_) –***

*pA_i_* is the same as in the *spaD* model, but in this model d = 0 and it’s fixed (we are not optimizing it). w_i_ and *g* are free parameters. See equations 6-8 in Bergert & Nosovsky (2007).


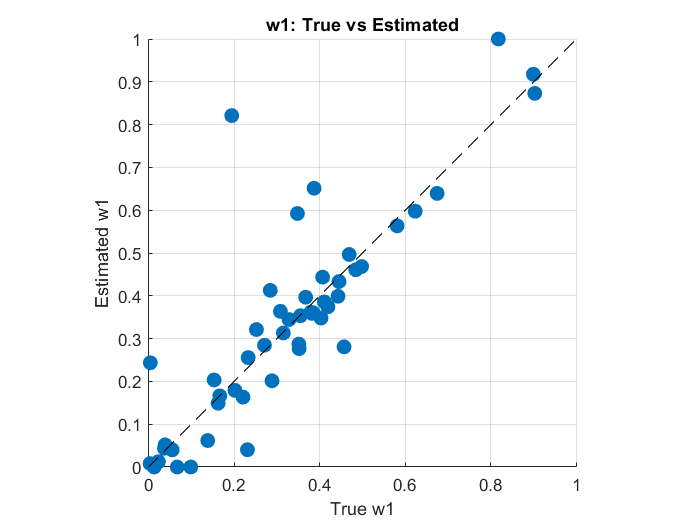

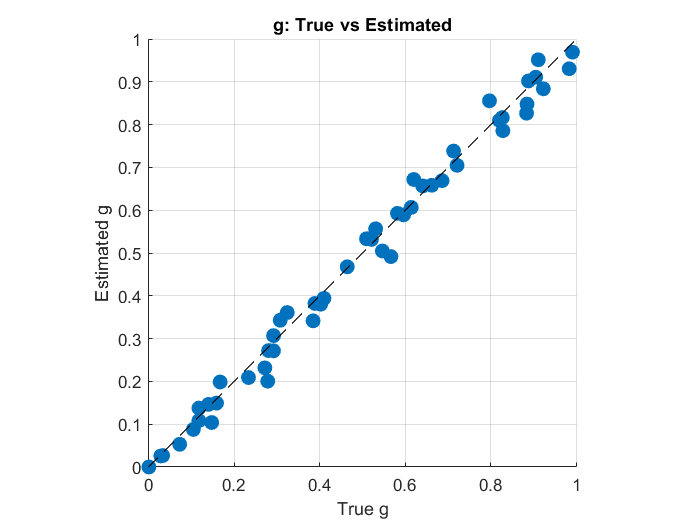
**Parameters recovery:**


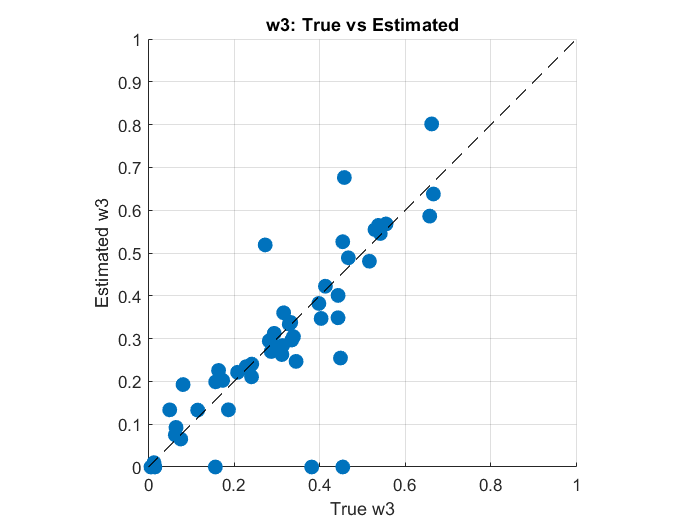

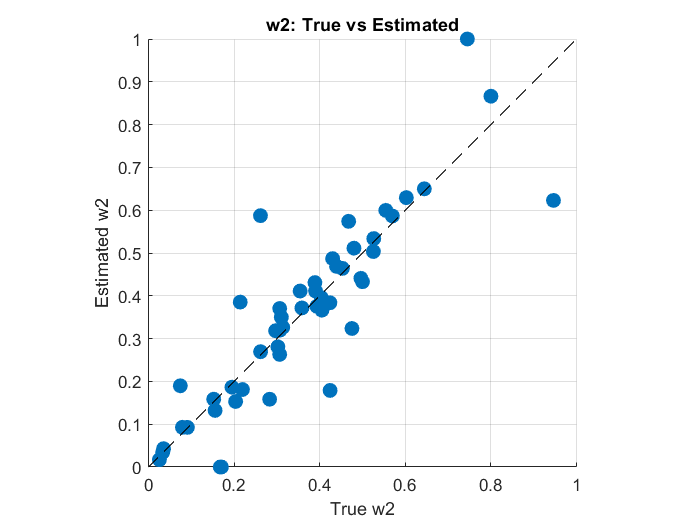


The correlation between estimated g and true g – .99

The correlation between estimated w_1_ and true w_1_ – .86

The correlation between estimated w_2_ and true w_2_ – .88

The correlation between estimated w_3_ and true w_3_ – .83

***TTB model (g) –***

This model is the deterministic *TTB* model; that is, the decision is made based on the first decisive attribute, ordered from the most to the least important attribute. For compatibility with the other models (and the work done by Bergert & Nosofsky, 2007) we added the guessing parameter, g, to the model. Thus, P(A|AB) = 1 if A on attribute i is better than B; otherwise, 0. We used the same nLL function as with the other models; within the nLL formula, $p_{i}=\frac{g}{2}+\left( 1-g \right)*{pA}_{i}$ is the same as before, but in this model *pA_i_* is equal to 1 (for A) or 0 (for B).

**Parameter recovery:**


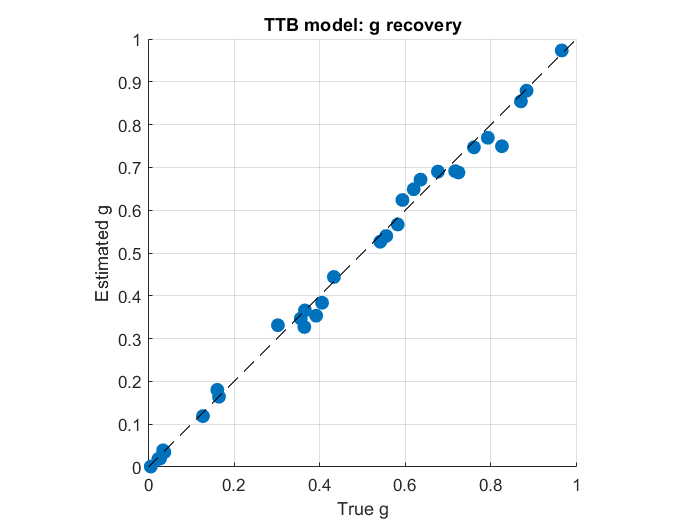


The correlation between estimated g and true g – .99

**Table I***Classification and BIC (experiment 1 data – 3 attributes):*

| **Participant** | **Strategy** | **gTTB BIC** | **spaD BIC** | **WAV BIC** | **TTB BIC** |
| --- | --- | --- | --- | --- | --- |
| 1 | WAV (0.50) | 105 | **102** | 122 | 122 |
| 2 | TTB (0.24) | 93 | 118 | 134 | **82** |
| 3 | WAV (0.88) | 138 | **112** | 118 | 170 |
| 4 | WAV (0.56) | 167 | **155** | 160 | 167 |
| 5 | WAV (0.75) | 106 | 103 | **82** | 109 |
| 6 | TTB (0.14) | 41 | 72 | 89 | **33** |
| 7 | WAV (0.68) | 156 | **151** | 163 | 181 |
| 8 | TTB (0.44) | 94 | 113 | 97 | **88** |
| 9 | TTB (0.48) | **82** | 96 | 102 | 98 |
| 10 | WAV (0.88) | 132 | 122 | **115** | 158 |
| 11 | WAV (0.58) | 99 | 104 | **83** | 97 |
| 12 | WAV (0.75) | 128 | **115** | 124 | 158 |
| 13 | WAV (0.78) | 129 | 115 | **92** | 140 |
| 14 | WAV (0.52) | **114** | 119 | 118 | 121 |
| 15 | WAV (0.68) | 102 | 100 | **88** | 121 |
| 16 | WAV (0.82) | 149 | **123** | 152 | 178 |
| 17 | WAV (0.85) | 108 | 103 | **78** | 135 |
| 18 | TTB (0.35) | 88 | 109 | 102 | **78** |
| 19 | TTB (0.42) | 67 | 89 | 72 | **61** |
| 20 | WAV (0.68) | 95 | 92 | **73** | 115 |
| 21 | WAV (0.80) | 120 | 108 | **100** | 133 |
| 22 | WAV (0.72) | 127 | 126 | **106** | 146 |
| 23 | WAV (0.89) | 123 | **95** | 117 | 159 |
| 24 | TTB (0.48) | 98 | 112 | 102 | **97** |
| 25 | TTB (0.40) | 98 | 106 | 102 | **94** |
| 26 | WAV (1.00) | 156 | **130** | 151 | 188 |
| 27 | WAV (0.67) | 152 | **142** | 154 | 170 |
| 28 | WAV (0.73) | 125 | 105 | **86** | 125 |
| 29 | TTB (0.44) | 89 | 117 | 93 | **83** |
| 30 | WAV (0.83) | 143 | 133 | **125** | 168 |
| 31 | WAV (0.60) | 111 | 117 | **98** | 118 |
| 32 | TTB (0.46) | **107** | 116 | 119 | 111 |
| 33 | WAV (0.80) | 110 | 99 | **88** | 136 |
| 34 | TTB (0.45) | 148 | **142** | 161 | 153 |
| 35 | WAV (0.54) | 146 | **138** | 152 | 158 |
| 36 | WAV (0.62) | 121 | 122 | **110** | 133 |
| 37 | WAV (0.50) | 208 | **194** | 195 | 195 |
| 38 | TTB (0.44) | 102 | 123 | 109 | **101** |
| 39 | TTB (0.36) | 79 | 99 | 88 | **73** |
| 40 | TTB (0.48) | **114** | 128 | 129 | 125 |
| 41 | WAV (0.79) | 125 | 109 | **87** | 149 |
| 42 | WAV (0.64) | 131 | **120** | 132 | 150 |
| 43 | WAV (0.90) | 121 | 100 | **61** | 136 |
| 44 | WAV (0.65) | 117 | **112** | 115 | 137 |
| 45 | WAV (0.72) | 112 | 103 | **94** | 133 |
| 46 | WAV (0.61) | 120 | 121 | **115** | 133 |
| 47 | WAV (0.71) | 103 | 101 | **88** | 125 |
| 48 | TTB (0.48) | 108 | **107** | 121 | 117 |
| 49 | WAV (0.85) | 124 | 115 | **110** | 139 |
| 50 | WAV (0.74) | 143 | 135 | **132** | 156 |
| 51 | WAV (0.82) | 130 | 108 | **106** | 144 |
| 52 | TTB (0.29) | 74 | 90 | 98 | **67** |
| 53 | WAV (0.80) | 208 | **195** | 196 | 197 |
| 54 | TTB (0.38) | 121 | 129 | 121 | **110** |
| **Mean** |  | **118.65** | **116.85** | **113.43** | **129.09** |

*Note: The numbers in parentheses, below the classification, are the %WAV. The lowest BIC is in bold.*

**Table J***Classification and BIC (experiment 1 data – 4 attributes):*

| **Participant** | **Strategy** | **gTTB BIC** | **spaD BIC** | **WAV BIC** | **TTB BIC** |
| --- | --- | --- | --- | --- | --- |
| 1 | TTB (0.44) | **133** | **133** | 143 | **133** |
| 2 | TTB (0.35) | 114 | 129 | 140 | **107** |
| 3 | WAV (0.77) | 159 | **146** | 152 | 188 |
| 4 | WAV (0.55) | 208 | **192** | 196 | 198 |
| 5 | WAV (0.62) | 133 | **122** | 127 | 148 |
| 6 | TTB (0.43) | 91 | 107 | 103 | **90** |
| 7 | WAV (0.54) | 159 | **148** | 151 | 156 |
| 8 | TTB (0.3) | 138 | 151 | 170 | **134** |
| 9 | WAV (0.66) | 125 | 127 | **98** | 133 |
| 10 | WAV (0.79) | 154 | 136 | **126** | 168 |
| 11 | WAV (0.53) | 130 | 137 | **126** | 134 |
| 12 | WAV (0.88) | 158 | **141** | 151 | 191 |
| 13 | TTB (0.48) | **92** | 115 | 111 | 107 |
| 14 | WAV (0.5) | **79** | 106 | 93 | 93 |
| 15 | WAV (0.79) | 132 | 132 | **111** | 178 |
| 16 | WAV (0.75) | 165 | **146** | 157 | 184 |
| 17 | WAV (0.71) | 127 | 119 | **102** | 146 |
| 18 | TTB (0.43) | 119 | 131 | 123 | **111** |
| 19 | TTB (0.35) | **89** | 129 | 123 | **89** |
| 20 | WAV (0.52) | **110** | 116 | 119 | 123 |
| 21 | WAV (0.56) | 144 | **136** | 145 | 156 |
| 22 | WAV (0.61) | 141 | **116** | 126 | 146 |
| 23 | WAV (0.92) | **135** | 141 | 146 | 193 |
| 24 | TTB (0.38) | **108** | 127 | 140 | 115 |
| 25 | WAV (0.56) | **136** | 140 | **136** | 148 |
| 26 | WAV (0.8) | 163 | **147** | 152 | 196 |
| 27 | WAV (0.59) | 175 | **153** | 174 | 183 |
| 28 | TTB (0.49) | 139 | 141 | 139 | **136** |
| 29 | TTB (0.29) | 104 | 124 | 140 | **93** |
| 30 | WAV (0.73) | 163 | **149** | 165 | 192 |
| 31 | TTB (0.49) | **113** | 119 | 126 | 123 |
| 32 | WAV (0.72) | 153 | 137 | **115** | 158 |
| 33 | WAV (0.74) | 169 | **157** | 163 | 190 |
| 34 | TTB (0.27) | 150 | 159 | 174 | **137** |
| 35 | WAV (0.63) | 160 | 154 | **133** | 157 |
| 36 | TTB (0.48) | **144** | 153 | 152 | 149 |
| 37 | WAV (0.64) | 198 | **185** | 186 | 196 |
| 38 | WAV (0.64) | 141 | 134 | **118** | 145 |
| 39 | TTB (0.25) | 95 | 131 | 140 | **84** |
| 40 | TTB (0.38) | 97 | 131 | 115 | **89** |
| 41 | WAV (0.58) | 122 | **114** | 126 | 143 |
| 42 | WAV (0.67) | 171 | **144** | 155 | 177 |
| 43 | WAV (0.69) | 114 | 116 | **103** | 126 |
| 44 | WAV (0.67) | 134 | 133 | **126** | 161 |
| 45 | WAV (0.7) | 165 | **148** | **148** | 182 |
| 46 | TTB (0.34) | **119** | 137 | 152 | **119** |
| 47 | WAV (0.5) | **122** | 135 | 126 | 126 |
| 48 | WAV (0.52) | 122 | 132 | **118** | 122 |
| 49 | WAV (0.8) | 138 | **127** | 139 | 188 |
| 50 | WAV (0.75) | 147 | **138** | 159 | 191 |
| 51 | WAV (0.63) | 155 | 150 | **149** | 167 |
| 52 | TTB (0.33) | **106** | 124 | 158 | 111 |
| 53 | WAV (0.64) | 209 | 201 | **189** | 191 |
| 54 | WAV (0.52) | 189 | 197 | **179** | 181 |
| **Mean** |  | **138.0741** | **138.7593** | **139.5185** | **147.8148** |

*Note: The numbers in parentheses, below the classification, are the %WAV. The lowest BIC is in bold.*

**Table K***Classification and BIC (experiment 1 data – 5 attributes):*

| **Participant** | **Strategy** | **gTTB BIC** | **spaD BIC** | **WAV BIC** | **TTB BIC** |
| --- | --- | --- | --- | --- | --- |
| 1 | TTB (0.58) | 136 | 136 | **126** | 146 |
| 2 | TTB (0.19) | 97 | 129 | 146 | **84** |
| 3 | TTB (0.74) | 180 | **154** | 158 | 193 |
| 4 | TTB (0.62) | 219 | **197** | 198 | 206 |
| 5 | TTB (0.59) | 189 | **171** | 176 | 189 |
| 6 | TTB (0.67) | 103 | **88** | 90 | 121 |
| 7 | TTB (0.76) | 188 | 173 | **170** | 196 |
| 8 | TTB (0.55) | 159 | 156 | **143** | 152 |
| 9 | TTB (0.64) | 123 | 112 | **107** | 134 |
| 10 | TTB (0.91) | 166 | 156 | **140** | 201 |
| 11 | TTB (0.59) | 133 | 143 | **119** | 143 |
| 12 | TTB (0.71) | 176 | **139** | 155 | 188 |
| 13 | TTB (0.60) | 163 | **158** | 160 | 178 |
| 14 | TTB (0.56) | 143 | 143 | **134** | 149 |
| 15 | TTB (0.62) | 145 | 142 | **130** | 158 |
| 16 | TTB (0.57) | 198 | **187** | 193 | 200 |
| 17 | TTB (0.69) | 143 | 127 | **114** | 159 |
| 18 | TTB (0.34) | 118 | 144 | 149 | **111** |
| 19 | TTB (0.37) | 121 | 146 | 146 | **111** |
| 20 | TTB (0.50) | **112** | 125 | 115 | 115 |
| 21 | TTB (0.66) | **138** | 143 | 140 | 173 |
| 22 | TTB (0.65) | 150 | 140 | **126** | 155 |
| 23 | TTB (0.86) | 155 | **144** | 170 | 204 |
| 24 | TTB (0.44) | 108 | 130 | 123 | **107** |
| 25 | TTB (0.56) | 146 | 140 | **133** | 143 |
| 26 | TTB (0.67) | 170 | **150** | 161 | 179 |
| 27 | TTB (0.59) | 188 | 174 | **173** | 185 |
| 28 | TTB (0.45) | 107 | 132 | 118 | **106** |
| 29 | TTB (0.36) | 103 | 136 | 126 | **93** |
| 30 | TTB (0.89) | 159 | 148 | **137** | 190 |
| 31 | TTB (0.56) | 129 | 150 | **111** | 123 |
| 32 | TTB (0.45) | 153 | **146** | 158 | 149 |
| 33 | TTB (0.78) | 183 | 169 | **163** | 197 |
| 34 | TTB (0.38) | 133 | 145 | 152 | **126** |
| 35 | TTB (0.42) | 146 | 152 | 155 | **140** |
| 36 | TTB (0.62) | 164 | 165 | **152** | 175 |
| 37 | TTB (0.48) | 217 | 202 | 194 | **192** |
| 38 | TTB (0.47) | **123** | 138 | 134 | 126 |
| 39 | TTB (0.26) | 122 | 158 | 168 | **111** |
| 40 | TTB (0.39) | 120 | 140 | 149 | **119** |
| 41 | TTB (0.63) | 130 | 129 | **107** | 140 |
| 42 | TTB (0.76) | 182 | 156 | **152** | 199 |
| 43 | TTB (0.43) | **103** | 128 | 134 | 119 |
| 44 | TTB (0.37) | **95** | 136 | 133 | 102 |
| 45 | TTB (0.45) | 143 | **141** | 151 | 142 |
| 46 | TTB (0.50) | **123** | 144 | 158 | 158 |
| 47 | TTB (0.55) | 131 | 136 | **115** | 126 |
| 48 | TTB (0.49) | **143** | 149 | 146 | **143** |
| 49 | TTB (0.77) | 167 | **151** | 157 | 191 |
| 50 | TTB (0.80) | 168 | **161** | 170 | 200 |
| 51 | TTB (0.49) | **141** | 147 | 155 | 152 |
| 52 | TTB (0.38) | 116 | 149 | 126 | **103** |
| 53 | TTB (0.44) | 221 | 203 | 200 | **196** |
| 54 | TTB (0.50) | 173 | **160** | 168 | 168 |
| **Mean** |  | **147.4444** | **148.4815** | **146** | **153.0741** |

*Note: The numbers in parentheses, below the classification, are the %WAV. The lowest BIC is in bold.*
